# Supplementary material for: Magmatic and thermally produced reactive phosphorus 3.2 billion years ago and its implications for early life
Source: Commun Earth Environ. 2025 Nov 13;6(1):895. doi: 10.1038/s43247-025-02824-x (PMC12615253; doi:10.1038/s43247-025-02824-x)
Supplement: Supplementary file 2 — Supplementary Material [file 43247_2025_2824_MOESM2_ESM.pdf]

Supplementary Materials for

Magmatic phosphite and thermally polymerized phosphate 3.2 billion years ago and its implications for early life

Abu Saeed Baidya[a*], Michelle M. Gehringer[b], Cristian Savaniu[c], Christoph Heubeck[d], Eva E. Stüeken[a]

[a]School of Earth and Environmental Sciences, University of St Andrews, Queen's Terrace, St Andrews KY16 9TS, United Kingdom

[b]Department of Microbiology, University of Kaiserslautern-Landau (RPTU), Gottlieb-Daimler Str., 67663 Kaiserslautern, Germany

[c]School of Chemistry, University of St Andrews, N Haugh, St Andrews KY16 9ST, United Kingdom

[d]Department of Geosciences, Friedrich-Schiller University Jena, Burgweg 11, 07749 Jena, Germany

* Corresponding Author: Abu Saeed Baidya (asb27@st-andrews.ac.uk)

This PDF file includes:

Supplementary Notes
Figs. S1 to S7
Tables S1 to S6
References (1 to 3)

Other Supplementary Materials for this manuscript include the following:

Supplementary Data SD1 to SD3

Data SD1 - XRD data for all experimental products
Data SD2 - NMR data for all experimental products
Data SD3 - Additional Geological Maps

Supplementary Notes

Sedimentary strata of the Moodies Group occur throughout the ca. 110 km * 40 km Barberton Greenstone Belt (BGB), reach up to 3.7 km in thickness, and consist largely of sandstones deposited in a range of alluvial, fluvial, coastal and deltaic environments. Siltstones and shales are subordinate; jaspilites, BIFs, lava and tuffaceous strata are rare. All Moodies strata are strongly folded and offset by faulting; most strata dip subvertically. However, the metasedimentary units preserve sedimentary textures in excellent quality at the micro- and macroscale in many places.

The investigated rocks were sampled from borehole BASE-1A, drilled on the west-facing, overturned eastern limb of the Eureka Syncline, a major, boomerang-shaped structural element in the north-central BGB. The borehole targeted well-developed strata in the mid-section of the Moodies Group (Fig. 1). The drilled stratigraphic interval includes (base-up) a major prograding delta (unit MdS1 and MdQ2 of Anhaeusser [1]), abruptly overlain by the best stratigraphic marker in the BGB, a widespread amygdaloidal basaltic lava thought to separate a gradually subsiding, more-or-less uniform Moodies basin from an overlying stratigraphically compartmentalized, tectonically active Moodies basin. This unit is overlain by heterogenous siliciclastic sediment of coastal-plain, tidal, (pro-)deltaic and perhaps lacustrine facies.

In addition to the primary magmatic event shortly before 3.2 Ga (see main text), two younger events are relevant to the discussion: first, thermal alteration related to sulfidic and Au mineralization along deep-reaching, subvertical brittle-ductile fault zones occurred along the northern margin of the BGB at ca. 3084 Ma [2]. This event is not known to have been associated with major magmatism in the study area and is therefore unlikely to play a role in our interpretation. Second, a final episode of magmatism affected the region at ca. 2967 Ma when the Badplaas dike swarm of doleritic magma intruded the still young Kaapvaal craton [3], feeding the overlying Nsuze Group, the world's oldest rift basin fill. These dikes are numerous throughout the BGB and adjacent plutonic terrain. They are dominantly oriented NW-SE and weather recessively among the silica-rich sedimentary strata of the BGB. Although the dolerites are usually poorly exposed, they are readily mappable because they form deep linear valleys. In the study area, no such morphological feature is recognizable. In addition, the composition of the mafic magmatic intrusions encountered in borehole BASE-1A differs from the geochemically intermediate, feldspar-porphyritic composition of this dike swarm. It is therefore unlikely that the intrusions sampled in this study are related to this event.

Readers are referred to Supplementary data SD3 for additional geological maps.

Supplementary images and tables

The following section contains images and data tables.

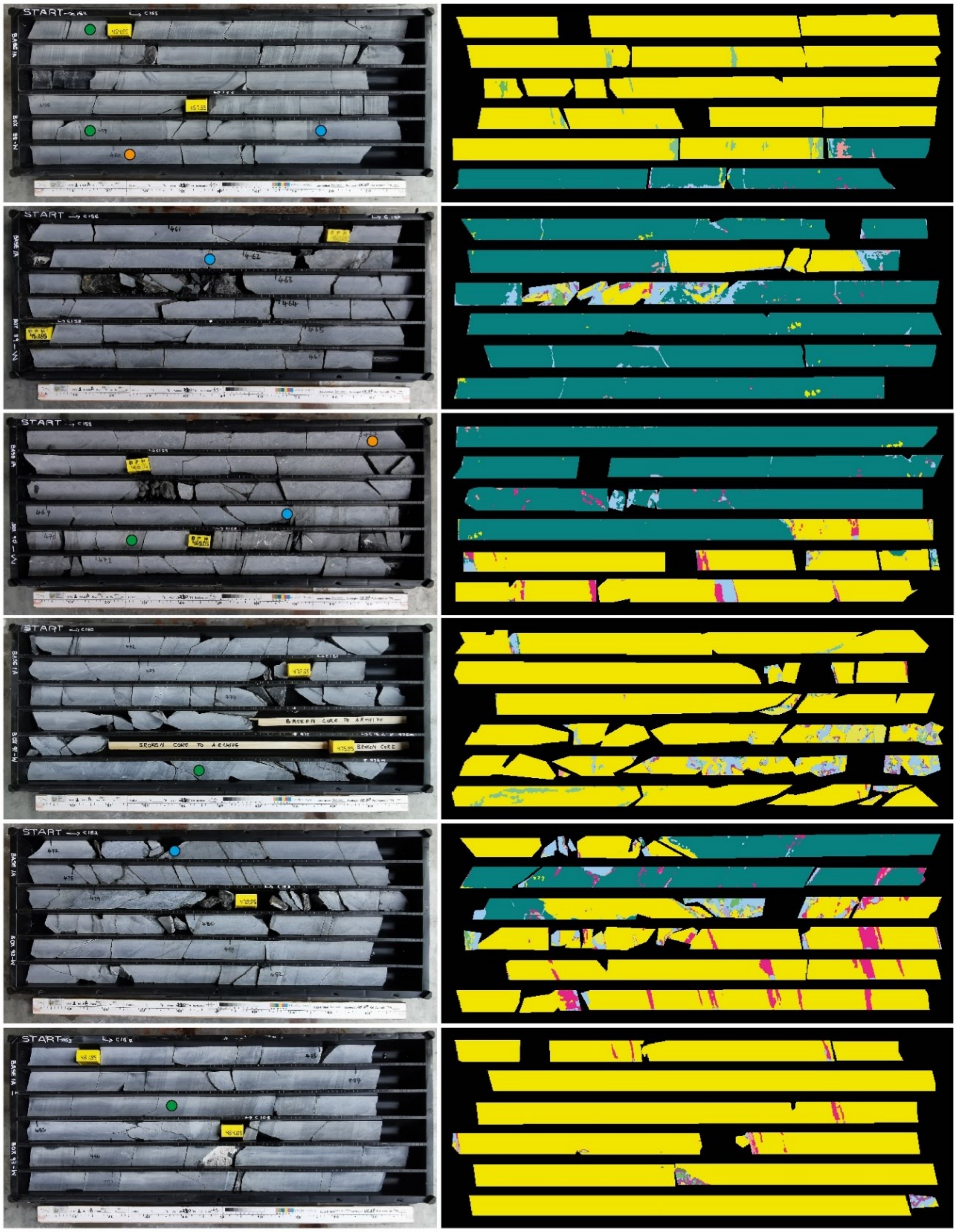

Figure S1: Photograph and corresponding spectral images of BASE-1A core trays. *The spectral images are flipped to the images of corresponding trays due to instrument settings. Green, blue, and orange circles in the left panel show locations of sedimentary, contact zones and of sampled intrusive rocks, respectively. Yellow and green colors in the hyperspectral images in the right panel represent sedimentary and intrusive rocks, respectively.*

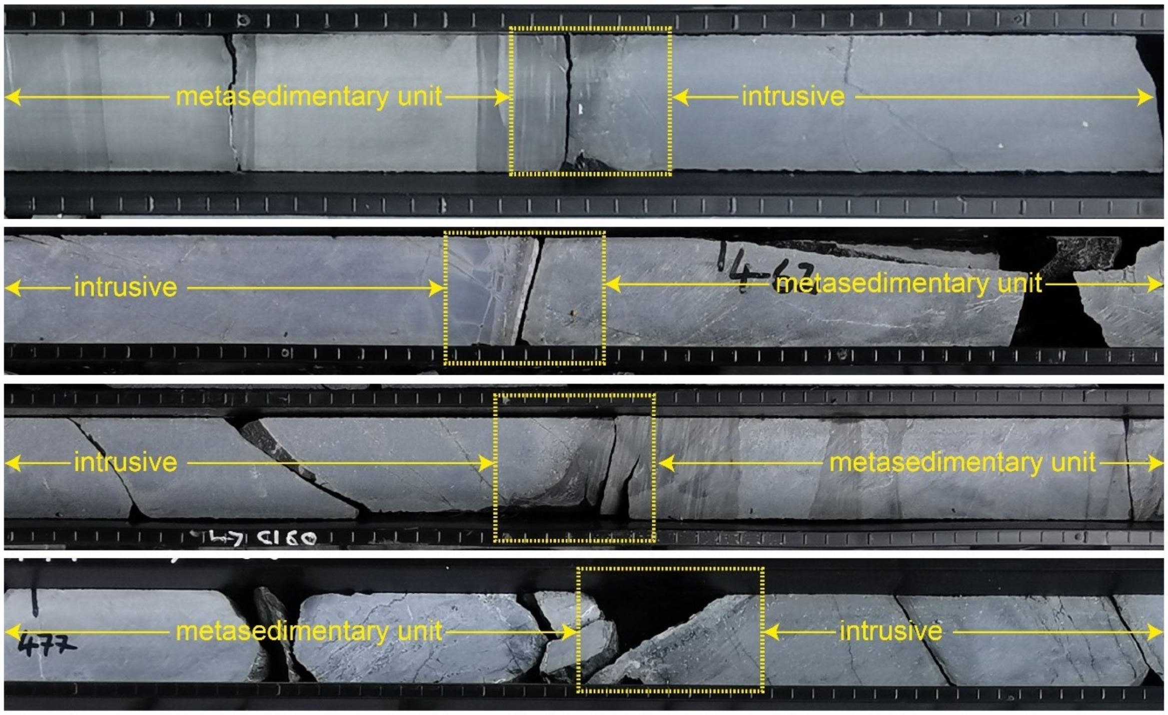

Figure S2: Detailed photographs of contact zones in the core samples. *Contact zones (from top to bottom panels are taken from Tray 1, 2, 3, and 5 in Fig. S1, respectively) are marked by yellow boxes. Tick marks of the core tray are spaced 1 cm apart.*

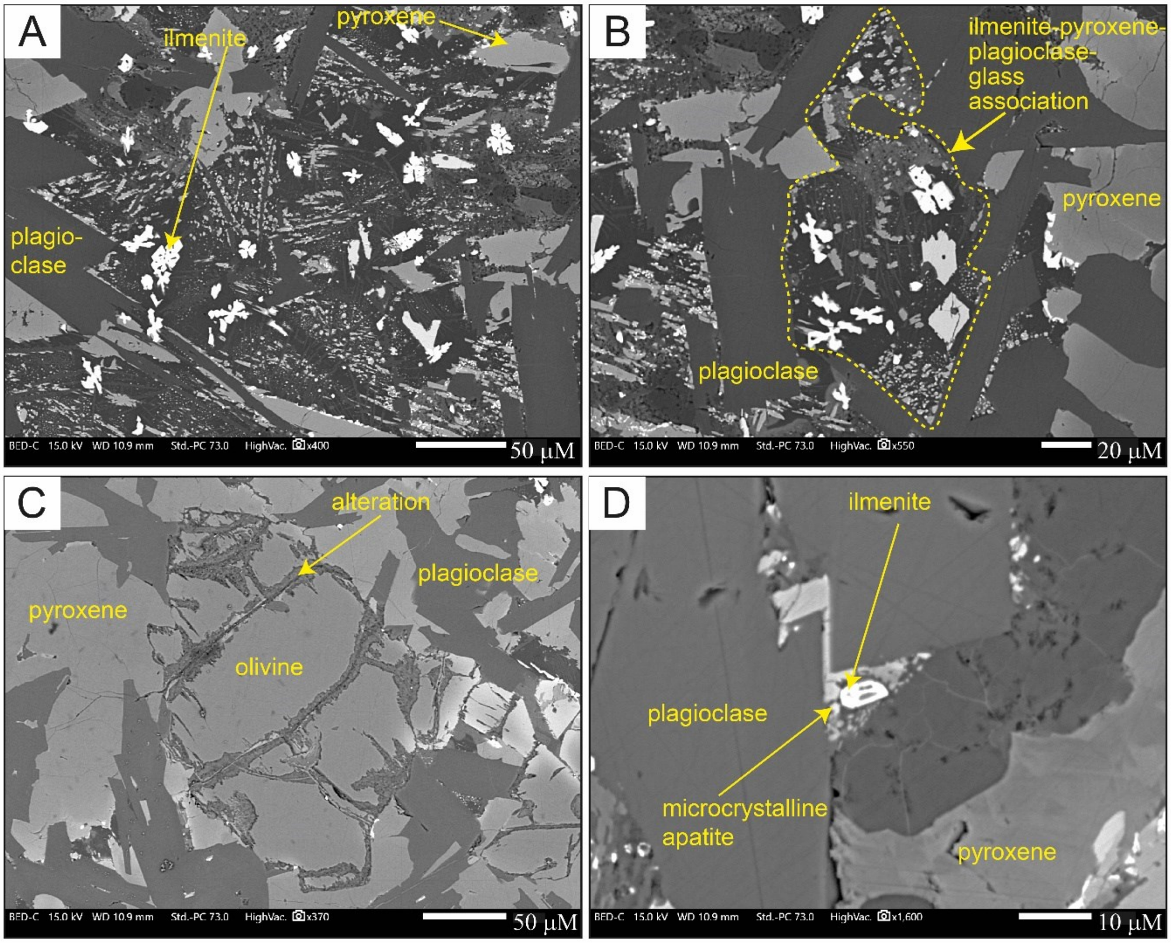

Figure S3: BSE images show key textures and mineralogy of intrusive units. *All four textures are present in sample no. B22. A shows that large pyroxene and plagioclase crystals are associated with skeletal ilmenite and fine-grained plagioclase and pyroxene. Darkest phase is most likely glass. B shows an association of plagioclase, pyroxene, skeletal ilmenite, and glass surrounded by large plagioclase and pyroxene grains. C shows minor alteration of olivine along the grain boundary and fractures. D shows microcrystalline apatite in association with skeletal ilmenite.*

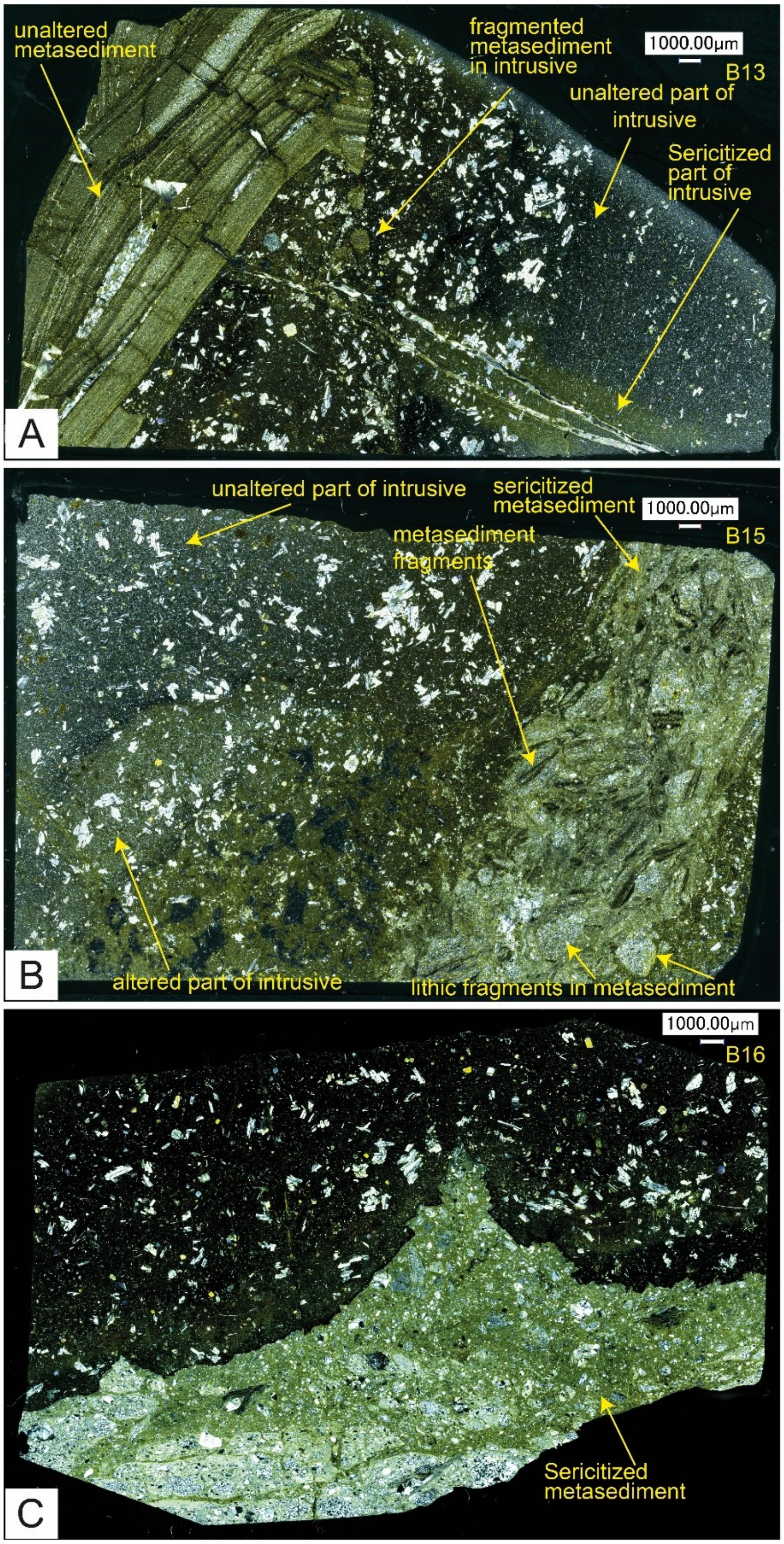

Figure S4: Cross-polarized microscopic images showing the nature of contacts between the intrusive and the metasedimentary strata. *A shows that the silty shale is layered and lacks alteration while the intrusive unit shows some alteration along the fractures. It also shows some*

fragments of the metasedimentary unit within the intrusive. B shows sericitization of the intrusive as well as of the brecciated metasedimentary unit. The latter consists of silty shale and quartz-rich fragments. C shows a contact between unaltered intrusive rock (above) and sericitized and brecciated siltstone (below).

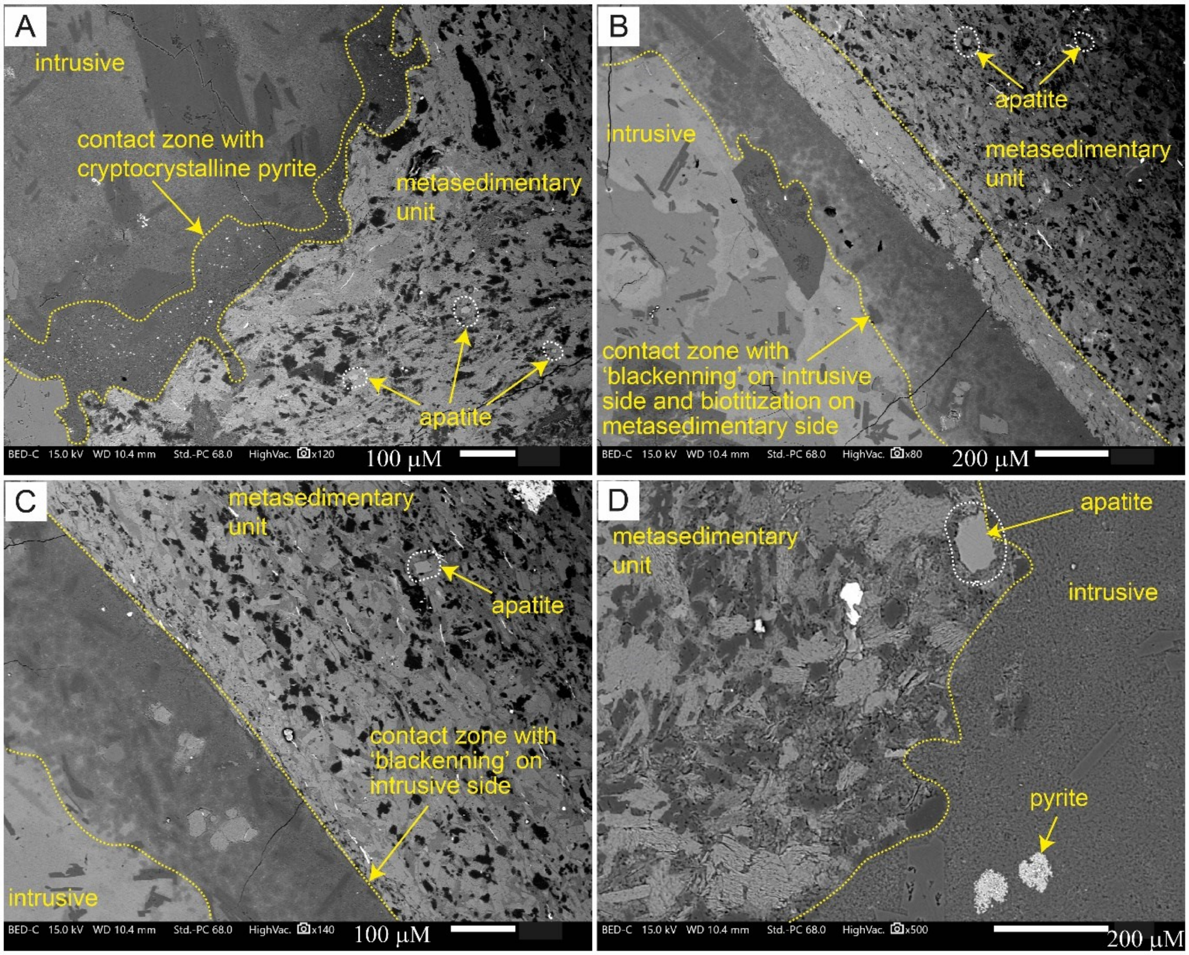

Figure S5: BSE images showing key textures of the contact zone between the intrusive and metasedimentary units. *All the textures are observed in sample B13. A shows the formation of cryptocrystalline pyrite along the contact zone between intrusive and metasedimentary unit. B shows the compositional change (blackening in BSE shades) in the intrusive side and biotite formation at the contact zone. C shows a similar compositional change in the intrusive side. D shows the presence of pyrite as microcrystalline aggregate near the contact zone. Apatite is common in the metasedimentary unit including at the contact boundary (D) and not observed in the intrusive unit.*

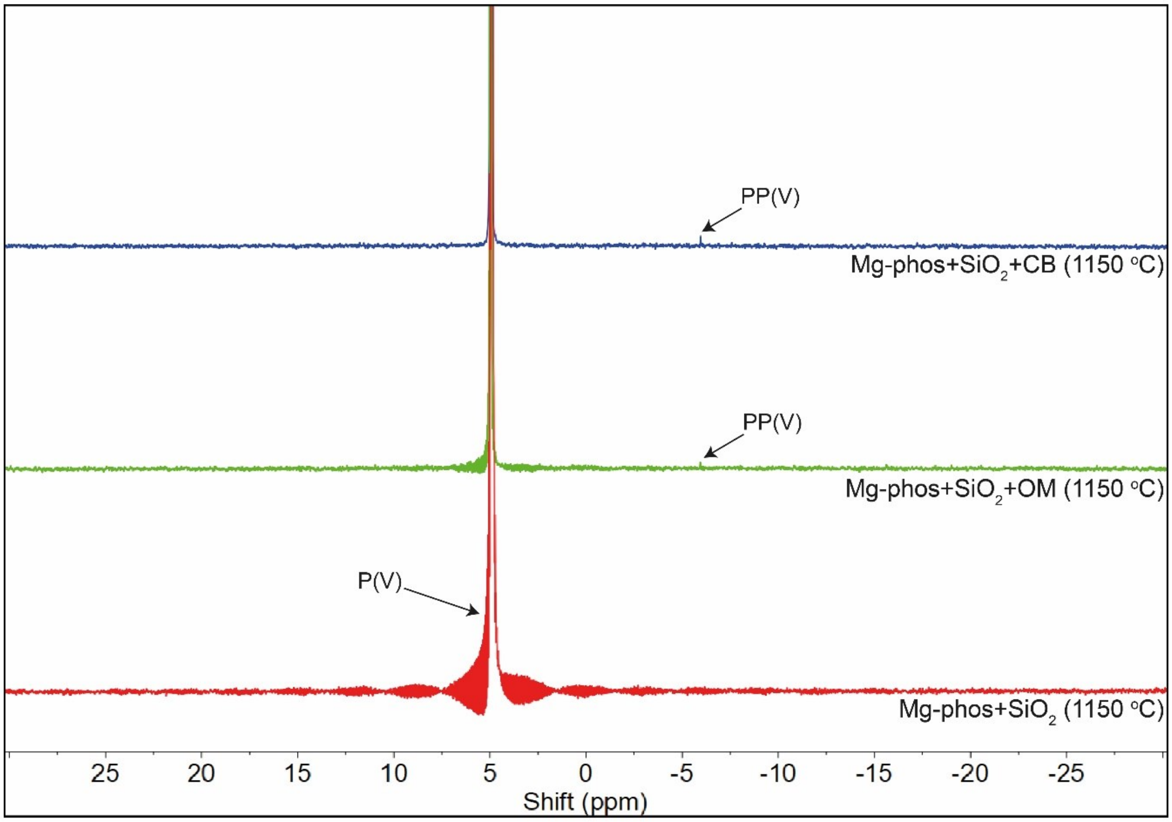

Figure S6: NMR patterns for the farringtonite experiments. PP(V) is detected in experiments with CB and OM but not in their absence.

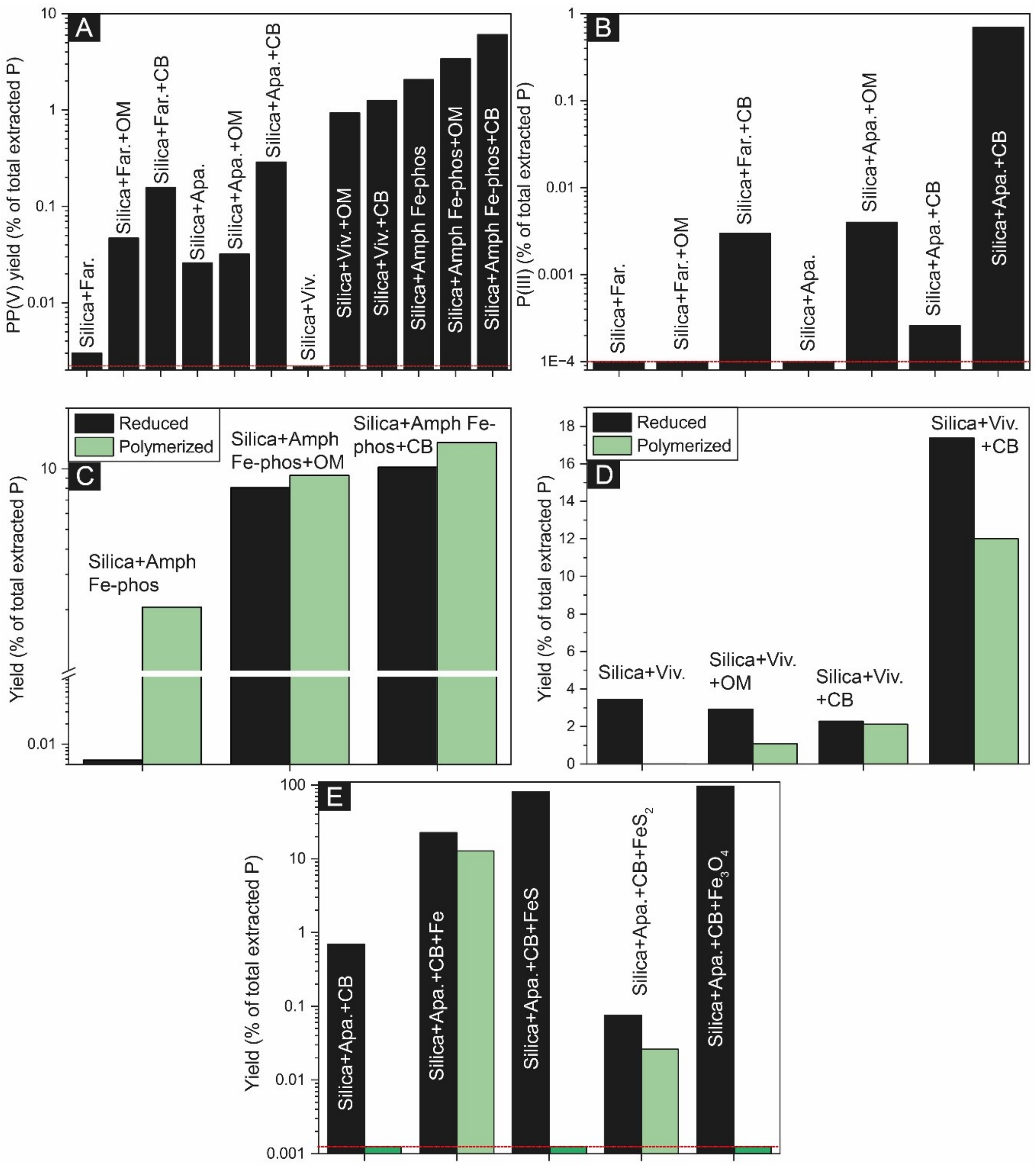

Figure S7: Experimental yield of reduced and polymerized P species from experiments. *The red lines show the detection limit of either IC-ICPMS or NMR. In A, B, C, the experiments were conducted at 1150 °C. In D, the silica+apatite+CB experiment with higher yield was conducted at 1300 °C, while the others were conducted at 1150 °C. In E, all experiments were conducted at 1300 °C.*

Table S1: Samples of BASE-1A core in stratigraphic order, following ICDP naming conventions (except Lab ID)

Lab ID	Box	Core	Section	Section Position [cm]	Core Depth [m]	Lithology
B20	88	154	5	15-20	454.8	siliciclastic metasediment
B21	88	156	2	15-20	459.0	siliciclastic metasediment
B13	88	156	2	74-79	459.6	contact
B22	88	156	3	20-25	460.1	intrusive basalt
B14	89	157	2	35-40	461.9	contact
B23	90	158	3	90-95	467.0	intrusive basalt
B15	90	159	3	63-68	469.7	contact
B24	90	159	4	25-30	470.2	siliciclastic metasediment
B25	91	162	2	25-30	476.4	siliciclastic metasediment
B16	92	162	3	32-37	477.3	contact
B26	93	164	3	30-35	484.5	siliciclastic metasediment

Table S2: TOC, Cr, Ti and total P in Moodies Group samples

Sample ID	Rock Types	P(ppm)	Cr (ppm)	Ti (%)	TOC [ppm]	St. dev TOC [ppm]
B20	Metasediment	330	723	0.331	863	11
B21	Metasediment	330	753	0.335	1872	
B24	Metasediment	310	794	0.356	1850	
B25	Metasediment	330	773	0.288	3310	28
B26	Metasediment	360	766	0.394	883	
B13	Contact zone	550	492	0.532	833	
B14	Contact zone	560	260	0.491	120	
B15	Contact zone	540	301	0.491	266	3
B16	Contact zone	370	376	0.393	343	1
B22	Intrusive	590	217	0.519	93	
B23	Intrusive	610	208	0.542	76	

Supplementary Table S3: P speciation in Moodies Group sample extracts

Samples	Concentration (ppb) in rock (as extracted in EDTA-NaOH)						Geometric Mean (ppb)			Mean + SD (ppb)			Mean – SD (ppb)		
	Avg P(III)	SD P(III)	Avg P(V)	SD P(V)	Avg PP(V)	SD PP(V)	P(III)	P(V)	PP(V)	P(III)	P(V)	PP(V)	P(III)	P(V)	PP(V)
Contact Zone															
B13	47.1	49.5	23157.6	5901.6	1784.6	3453.6	32.5	22503.7	185.0	85.2	30006.1	2120.7	12.4	16877.2	16.1
B14	55.5	60.7	28939.5	4549.9	451.7	647.9	35.4	28687.8	188.9	115.4	33807.0	958.4	10.8	24343.7	37.2
B15	54.5	27.9	25823.1	7722.0	266.6	325.9	48.4	25109.5	153.2	92.1	33391.7	553.8	25.4	18881.6	42.4
B16	12.0	0.7	14074.1	245.8	88.7	90.6	12.0	14073.0	61.3	12.7	14320.9	223.0	11.4	13829.4	16.9
Metasediments															
B20	40.4	8.6	8074.7	3118.8	11.6	1.3	39.9	7767.7	11.5	49.5	11545.2	12.9	32.2	5226.2	10.3
B21	10.8	2.3	6897.9	2263.0	12.5	0.1	10.7	6709.8	12.5	13.3	9371.9	12.5	8.6	4803.8	12.4
B24	6.3	1.2	6911.7	701.7	18.7	8.8	6.3	6893.9	17.7	7.6	7631.8	28.8	5.2	6227.3	10.8
B25	13.1	5.9	4676.0	1175.5	12.5	0.0	12.5	4601.5	12.5	19.8	5932.7	12.5	7.8	3569.1	12.5
B26	6.9	1.5	4544.6	1366.0	15.2	3.9	6.8	4440.8	15.0	8.5	6025.8	19.4	5.5	3272.7	11.6
Intrusive															
B22	23.9	15.4	32739.6	237.6	91.6	61.1	19.6	32739.0	76.2	45.7	32977.7	167.3	8.4	32502.0	34.7
B23	148.1	237.0	31534.5	3398.9	86.0	35.4	53.2	31397.2	81.2	268.3	34976.5	118.6	10.6	28184.1	55.6

Supplementary Table S4: Estimated concentrations in the Moodies samples

Extracted and estimated concentrations of different P species in the Moodies Group						
**Samples**	**Total P (ppm)**	**Total ext. P (ppm)**	**Extraction yield (%)**	**Estimated concentration in solids**		
				**Avg P(III) (ppm)**	**Avg P(V) (ppm)**	**Avg PP(V) (ppm)**
Contact zone						
B13	550	24.99	4.54	1.04	509.69	39.28
B14	560	29.45	5.26	1.06	550.35	8.59
B15	540	26.14	4.84	1.13	533.37	5.51
B16	370	14.17	3.83	0.31	367.37	2.32
**Metasedimentary**						
B20	330	8.13	2.46	1.64	327.89	0.47
B21	330	6.92	2.10	0.51	328.89	0.60
B24	310	6.94	2.24	0.28	308.88	0.84
B25	330	4.70	1.42	0.92	328.20	0.88
B26	360	4.57	1.27	0.54	358.26	1.20
Intrusive						
B22	590	32.86	5.57	0.43	587.93	1.64
B23	610	31.77	5.21	2.84	605.50	1.65

Supplementary Table S5: Summary of initial chemistry and experiment steps

Exp No.	Chemistry	Temp (^oC)	Weighted Mass (g)			
			Powder SiO_2	P source	C source	Fe-source
1	SiO_2	1150	0.60	None	None	None
2	SiO_2+Mg-phos	1150	0.42	0.18	None	None
3	SiO_2+Apatite	1150	0.42	0.18	None	None
4	SiO_2+ vivianite	1150	0.42	0.18	None	None
4un	SiO_2+vivianite	25	0.42	0.18	None	None
5	SiO_2+Amph. Fe-phos.	1150	0.42	0.18	None	None
6A	SiO_2+OM	1150	0.42	None	0.18	None
6B	SiO_2+CB	1150	0.42	None	0.18	None
7	SiO_2+Mg-phos.+OM	1150	0.24	0.18	0.18	None
8	SiO_2+Apatite+OM	1150	0.24	0.18	0.18	None
9	SiO_2+vivianite+OM	1150	0.24	0.18	0.18	None
10	SiO_2+ Amph. Fe-phos.+OM	1150	0.24	0.18	0.18	None
11	SiO_2+Mg-phosphate+CB	1150	0.24	0.18	0.18	None
12A	SiO_2+Apatite+CB	1150	0.24	0.18	0.18	None
12B	SiO_2+Apatite+CB	1150	0.24	0.18	0.18	None
13A	SiO_2+vivianite+CB	1150	0.24	0.18	0.18	None
13B	SiO_2+vivianite+CB	1150	0.24	0.18	0.18	None
14	SiO_2+Amph. Fe-phos. + CB	1150	0.24	0.18	0.18	None
15	SiO_2+Apatite+CB	1300	0.24	0.18	0.18	None
16A	SiO_2+vivianite+CB	1300	0.24	0.18	0.18	None
16B	SiO_2+vivianite+CB	1300	0.24	0.18	0.18	None
17	SiO_2+Apatite+CB+Fe	1300	0.14	0.18	0.18	0.10
18A	SiO_2+Apatite+CB+FeS	1300	0.08	0.18	0.18	0.16
18B	SiO_2+Apatite+CB+FeS	1300	0.06	0.18	0.18	0.16
19	SiO_2+Apatite+CB+FeS_2	1300	0.02	0.18	0.18	0.22
20A	SiO_2+Apatite+CB+Fe_3O_4	1300	0.10	0.18	0.18	0.14
20B	SiO_2+Apatite+CB+Fe_3O_4	1300	0.10	0.18	0.18	0.14

Notes: ‘Un’ is the abbreviation of ‘unheated’. A and B under the same experiment number are repeat experiments with the same initial composition.

Supplementary Table S6: Phosphorus speciation in the experimental products

Exp No.	Initial Chemistry	Temperature (°C)	P-C(III)	P(I)	P(III)	PP(IV)	P(V)	PP(V)	PPP(V)	PPPc(V)	PPPP(V)
1	SiO_2	1150	ND	ND	ND	ND	3.242	ND	ND	ND	ND
2	SiO_2+Mg-phosphate	1150	ND	ND	ND	ND	2069.474	0.053	ND	ND	ND
3	SiO_2+Apatite	1150	ND	ND	ND	ND	815.960	0.213	ND	ND	ND
4	SiO_2+ vivianite	1150	ND	ND	0.003	ND	0.043	ND	ND	ND	ND
4*	SiO_2+vivianite	25	ND	0.004	0.010	ND	0.513	ND	ND	ND	ND
5	SiO_2+ Amph Fe-phosphate	1150	ND	ND	0.002	ND	30.234	0.638	ND	ND	ND
6OM	SiO_2+OM	1150	ND	ND	ND	ND	20.537	0.378	ND	ND	ND
6CB*	SiO_2+CB	25	ND	ND	0.038	ND	0.149	0.029	ND	ND	ND
6CB	SiO_2+CB	1150	ND	0.004	0.049	ND	7.571	2.248	0.303	ND	ND
7	SiO_2+Mg-phosphate+OM	1150	ND	ND	ND	ND	456.963	0.213	ND	ND	ND
8	SiO_2+Apatite+OM	1150	ND	ND	0.011	ND	277.698	0.088	ND	ND	ND
9	SiO_2+vivianite+OM	1150	ND	0.009	0.967	1.585	82.677	0.817	0.132	ND	ND
10	SiO_2+ Amph Fe-phosphate+OM	1150	0.137	1.989	12.593	ND	150.399	6.224	3.925	0.903	5.854
11	SiO_2+Mg-phosphate+CB	1150	ND	ND	0.008	ND	244.525	0.384	ND	ND	ND
12A	SiO_2+Apatite+CB	1150	ND	0.006	0.001	ND	432.761	1.254	ND	ND	ND
12B	SiO_2+Apatite+CB	1150	ND	0.023	0.145	ND	14.880	0.734	ND	ND	ND
13A	SiO_2+vivianite+CB	1150	ND	ND	0.566	0.193	31.776	0.418	0.136	ND	0.152
13B	SiO_2+vivianite+CB	1150	0.460	2.312	16.382	3.337	29.365	8.509	5.785	1.033	8.465
14	SiO_2+ Amph Fe-phosphate+CB	1150	0.568	2.455	12.922	ND	118.698	9.503	3.521	0.745	7.356
15	SiO_2+Apatite+CB	1300	ND	0.002	0.015	ND	2.500	ND	ND	ND	ND
16A	SiO_2+vivianite+CB	1300	0.574	1.290	7.297	2.482	70.079	2.717	1.648	ND	3.041
16B	SiO_2+vivianite+CB	1300	0.812	2.170	9.524	3.294	44.592	3.772	2.972	ND	4.700
17	SiO_2+Apatite+CB+Fe	1300	0.227	0.137	3.525	0.248	11.513	1.202	0.351	ND	0.773
18A	SiO_2+Apatite+CB+FeS	1300	ND	ND	1.123	ND	0.363	ND	ND	ND	ND
18B	SiO_2+Apatite+CB+FeS	1300	ND	0.020	0.448	ND	ND	ND	ND	ND	ND
19	SiO_2+Apatite+CB+FeS2	1300	ND	0.249	0.194	ND	584.605	0.153	ND	ND	ND
20A	SiO_2+Apatite+CB+Fe3O4	1300	0.101	0.186	31.478	ND	0.520	ND	ND	ND	ND
20B	SiO_2+Apatite+CB+Fe3O4	1300	0.196	0.125	6.783	ND	0.150	ND	ND	ND	ND

Notes: Concentrations (ppm) are in EDTA-NaOH extract; Blue data are from IC-ICPMS analysis and rest are from NMR analysis; ND- Not detected; A and B are replicate experiments done at different times. The detection limit for the IC-ICPMS analysis is 0.01 ppb, therefore, during % calculation some numbers of reduction yield are expected to be up to three decimal points.

Supplementary Table S7: Diffraction angles, 2θ(°) with corresponding D-spacing values (Å) and relative intensities (%) of the most intense peaks for the products identified in the XRD spectra (Fig. 6 and 7 in the main text)

**Compound**	Pdf no.	Difraction angle (°), D-spacing values (Å), and relative intensity (%)			
SiO_2	00-046-1045	26.6°/3.34 Å 100	20.8°/4.25 Å 16	50.14°/1.8 Å 13	36.5°/2.45 Å 9
Barringerite (Fe_2P)	00-051-0943	40.3°/2.24 Å 100	44.2°/2.05 Å 72	47.3°/1.92 Å 39	
Schreibersite (Fe_3P)	00-064-0239	41.04°/2.2 Å 100	42.83°/2.1 Å 56	45.8°/1.98 Å 82	44.5°/2.03 Å 40
Tridymite/cristobalite (SiO_2)	00-039-1425	22.1°/4.04 Å 100	31.5°/2.84 Å 9	36.1°/2.48 Å 13	28.4°/3.13 Å 9
Sinoite (Si_2N_2O)	00018-1171	20.1 °/4.44 Å 100	26.5°/3.36 Å 100	37.1°/2.42 Å 80	18.95°/4.7 Å 80
β-Si_3N_4	00-033-1160	27.1°/3.29 Å 100	33.7°/2.66 Å 99	36.0°/2.49 Å 93	23.4°/3.8 Å 35
Oldhamite (CaS)	01-071-4760	31.8°/2.81 Å 100	45.6°/1.98 Å 80	56.67°/1.6 Å 70	66.46°/1.4 Å 70
Hydroxyapatite [$Ca_{10}(PO_4)_6(OH)_2$]	00-055-0592	32.9°/2.71 Å 100	31.78°/2.8 Å 26	39.8°/2.26 Å 18	25.86°/3.4 Å 9
Pseudowollastonite ($Ca_3Si_3O_9$)	00-031-0300	27.5°/3.23 Å 100	27.7°/3.22 Å 100	31.75°/2.8 Å 70	36.7°/2.45 Å 70

Supplementary references

1. Anhaeusser, C. R. The geology of the Sheba Hills area of the Barberton Mountain Land, South Africa, with particular reference to the Eureka Syncline. *South African J. Geol.* **79**, 253–280 (1976).

2. de Ronde, C. E. J., Kamo, S., Davis, D. W., de Wit, M. J. & Spooner, E. T. C. Field, geochemical and U-Pb isotopic constraints from hypabyssal felsic intrusions within the Barberton greenstone belt, South Africa: Implications for tectonics and the timing of gold mineralization. *Precambrian Res.* **49**, 261–280 (1991).

3. Olsson, J. R., Söderlund, U., Klausen, M. B. & Ernst, R. E. U–Pb baddeleyite ages linking major Archean dyke swarms to volcanic-rift forming events in the Kaapvaal craton (South Africa), and a precise age for the Bushveld Complex. *Precambrian Res.* **183**, 490–500 (2010).
