# Supplementary material for: Magmatic and thermally produced reactive phosphorus 3.2 billion years ago and its implications for early life
Source: Commun Earth Environ. 2025 Nov 13;6(1):895. doi: 10.1038/s43247-025-02824-x (PMC12615253; doi:10.1038/s43247-025-02824-x)
Supplement: Supplementary file 3 — Description of Additional Supplementary Files [file 43247_2025_2824_MOESM3_ESM.pdf]

Description of Additional Supplementary Files

File name: Supplementary Data 1

Description: XRD data for all experimental products

File name: Supplementary Data 2

Description: NMR data for all experimental products

File name: Supplementary Data 3

Description: Additional Geological Maps
