# Supplementary material for: Magmatic and thermally produced reactive phosphorus 3.2 billion years ago and its implications for early life
Source: Commun Earth Environ. 2025 Nov 13;6(1):895. doi: 10.1038/s43247-025-02824-x (PMC12615253; doi:10.1038/s43247-025-02824-x)
Supplement: Supplementary file 6 — Supplementary Data SD3 [file 43247_2025_2824_MOESM6_ESM.zip › Data S3 - Additional Geological Maps.pptx]

## Slide 1
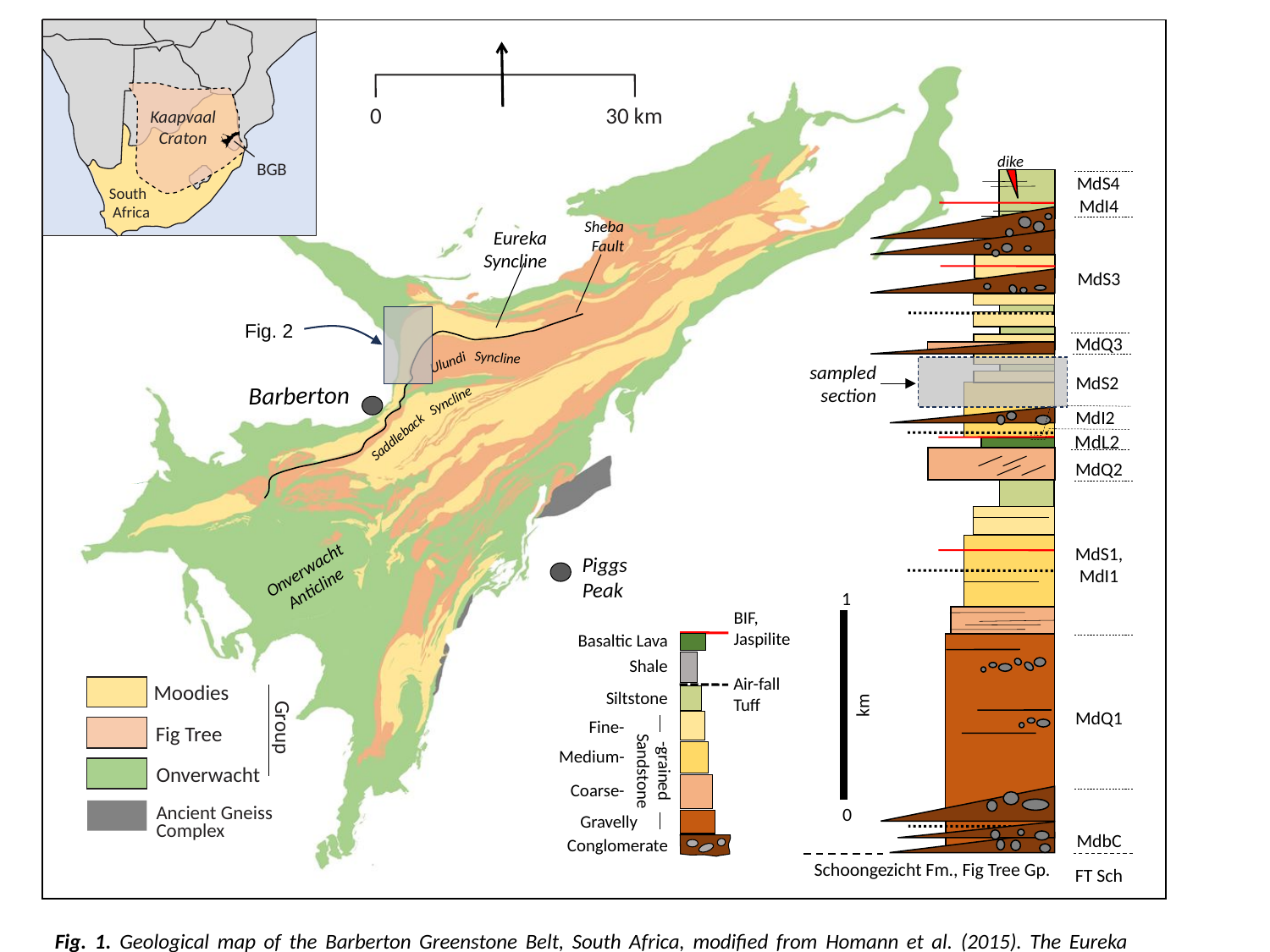

Kaapvaal
Craton
BGB
South
 Africa
30 km
0
dike
MdS4
MdI4
MdS3
MdQ3
MdS2
MdI2
MdL2
MdQ2
MdS1, MdI1
MdQ1
MdbC
FT Sch
1
km
0
Schoongezicht Fm., Fig Tree Gp.
sampled section
Sheba Fault
Eureka Syncline
Fig. 2
Syncline
Ulundi
Barberton
Syncline
Saddleback
Piggs Peak
Onverwacht Anticline
BIF, Jaspilite
Basaltic Lava
Shale
Air-fall Tuff
Siltstone
Fine-
Medium-
-grained
Sandstone
Coarse-
Gravelly
Conglomerate
Moodies
Group
Fig Tree
Onverwacht
Ancient Gneiss Complex
Fig. 1. Geological map of the Barberton Greenstone Belt, South Africa, modified from Homann et al. (2015). The Eureka Syncline is a large refolded structure in the north-central BGB. Stratigraphic column to the right shows generalized lithology of the Moodies Group in the Saddleback and Eureka Synclines which preserve the greatest stratigraphic thickness of this unit.

## Slide 2
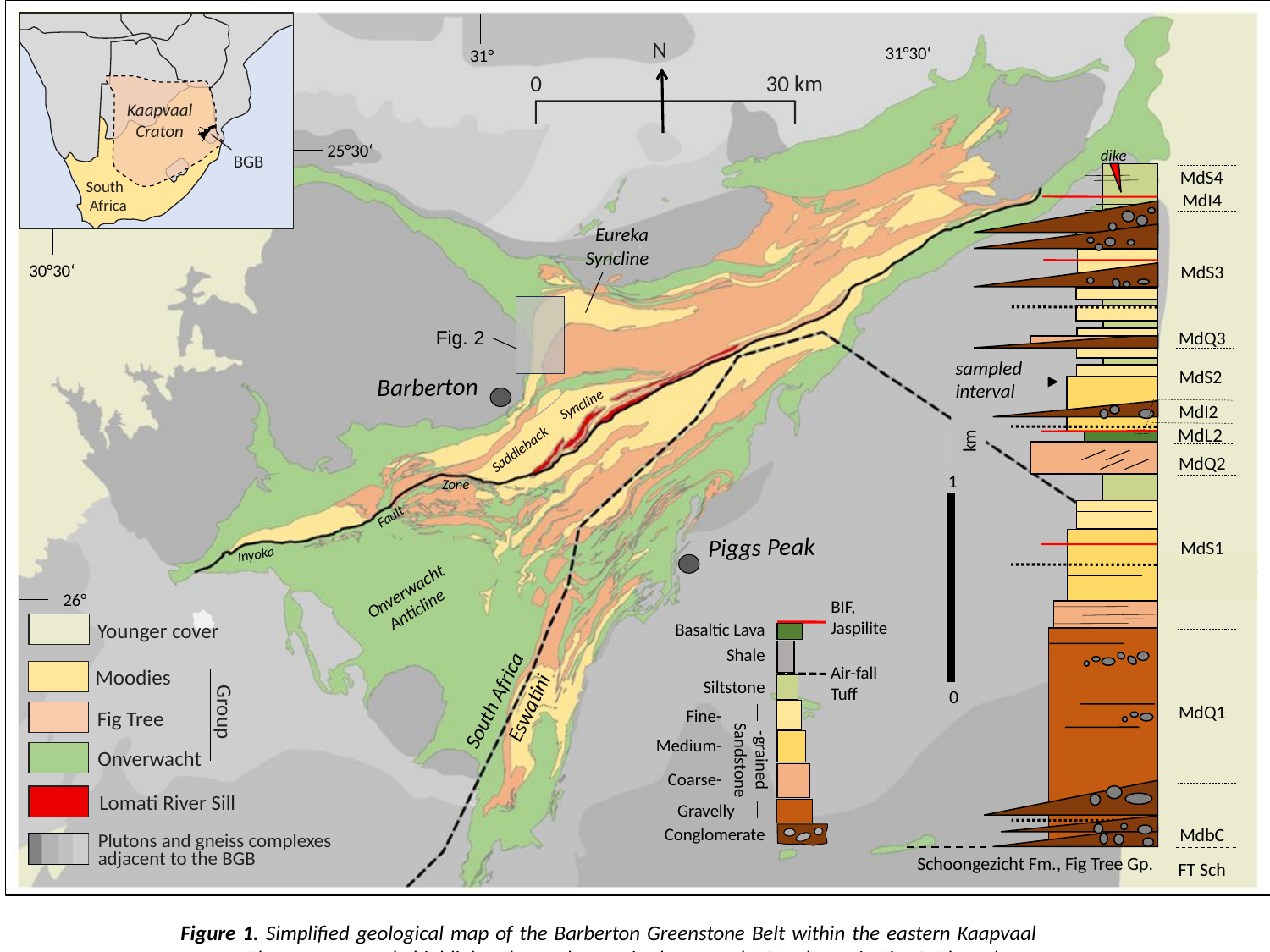

Kaapvaal
Craton
BGB
South
 Africa
N
30 km
0
31°30‘
31°
25°30‘
dike
MdS4
MdI4
MdS3
MdQ3
MdS2
MdI2
MdL2
MdQ2
MdS1
MdQ1
MdbC
FT Sch
km
1
0
Schoongezicht Fm., Fig Tree Gp.
Eureka Syncline
30°30‘
Fig. 2
sampled
interval
Barberton
Syncline
Saddleback
Zone
Fault
Piggs Peak
Inyoka
Onverwacht Anticline
26°
BIF, Jaspilite
Basaltic Lava
Shale
Air-fall Tuff
Siltstone
Fine-
Medium-
-grained
Sandstone
Coarse-
Gravelly
Conglomerate
Younger cover
Moodies
Group
Fig Tree
Onverwacht
Lomati River Sill
Plutons and gneiss complexes adjacent to the BGB
South Africa
Eswatini
Figure 1. Simplified geological map of the Barberton Greenstone Belt within the eastern Kaapvaal craton. The grey rectangle highlights the study area in the central BGB, shown in Fig. 2. The column to the right shows the generalized stratigraphy of the Moodies Group in the Saddleback Syncline, using the nomenclature of Anhaeusser (1976). Microbial mats and associated features occur in upper MdQ1.

## Slide 3
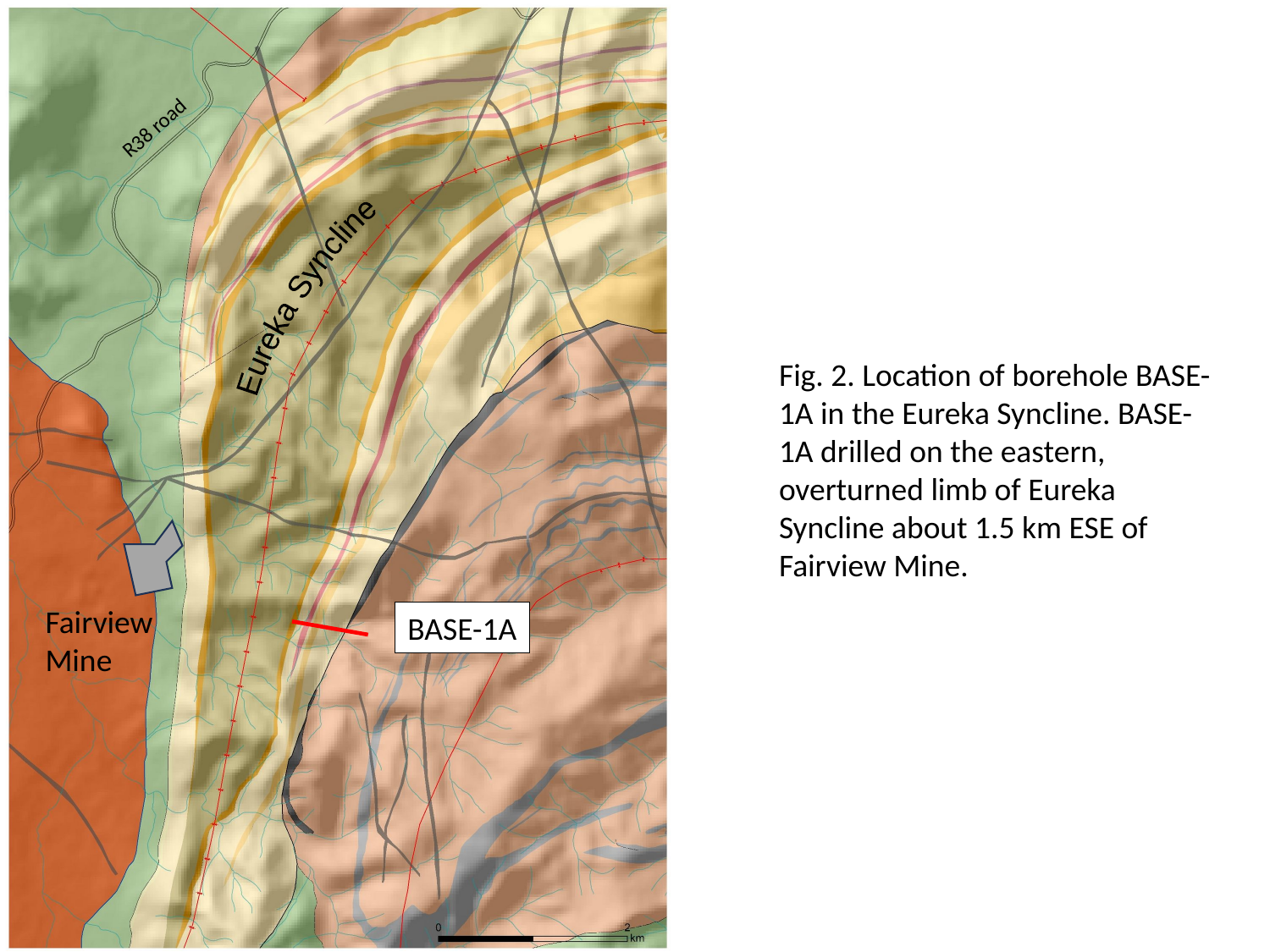

R38 road
Eureka Syncline
Fig. 2. Location of borehole BASE-1A in the Eureka Syncline. BASE-1A drilled on the eastern, overturned limb of Eureka Syncline about 1.5 km ESE of Fairview Mine.
Fairview
Mine
BASE-1A

## Slide 4
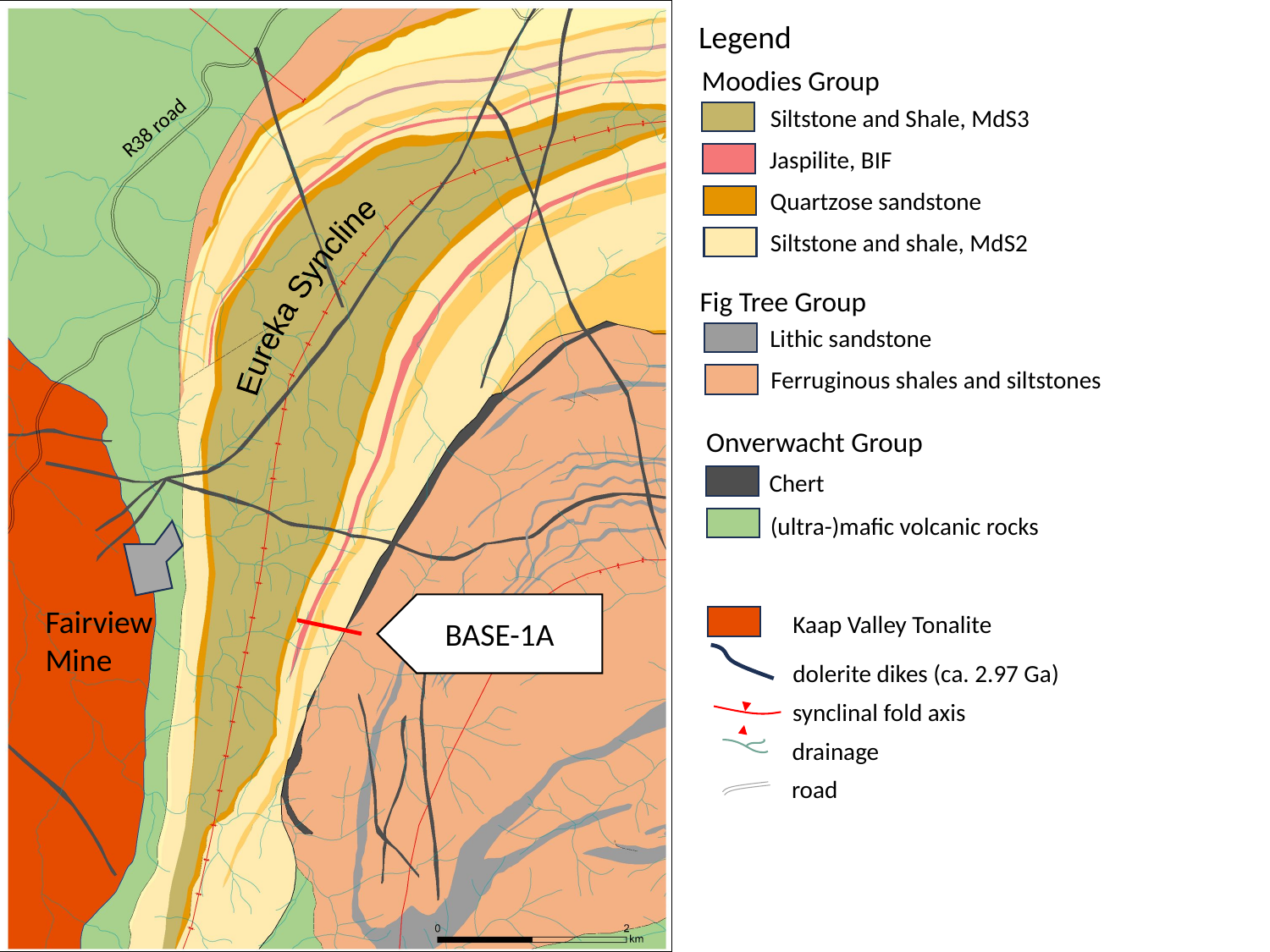

Legend
Moodies Group
Siltstone and Shale, MdS3
R38 road
Jaspilite, BIF
Quartzose sandstone
Siltstone and shale, MdS2
Eureka Syncline
Fig Tree Group
Lithic sandstone
Ferruginous shales and siltstones
Onverwacht Group
Chert
(ultra-)mafic volcanic rocks
BASE-1A
Fairview
Mine
Kaap Valley Tonalite
dolerite dikes (ca. 2.97 Ga)
synclinal fold axis
drainage
road

## Slide 5
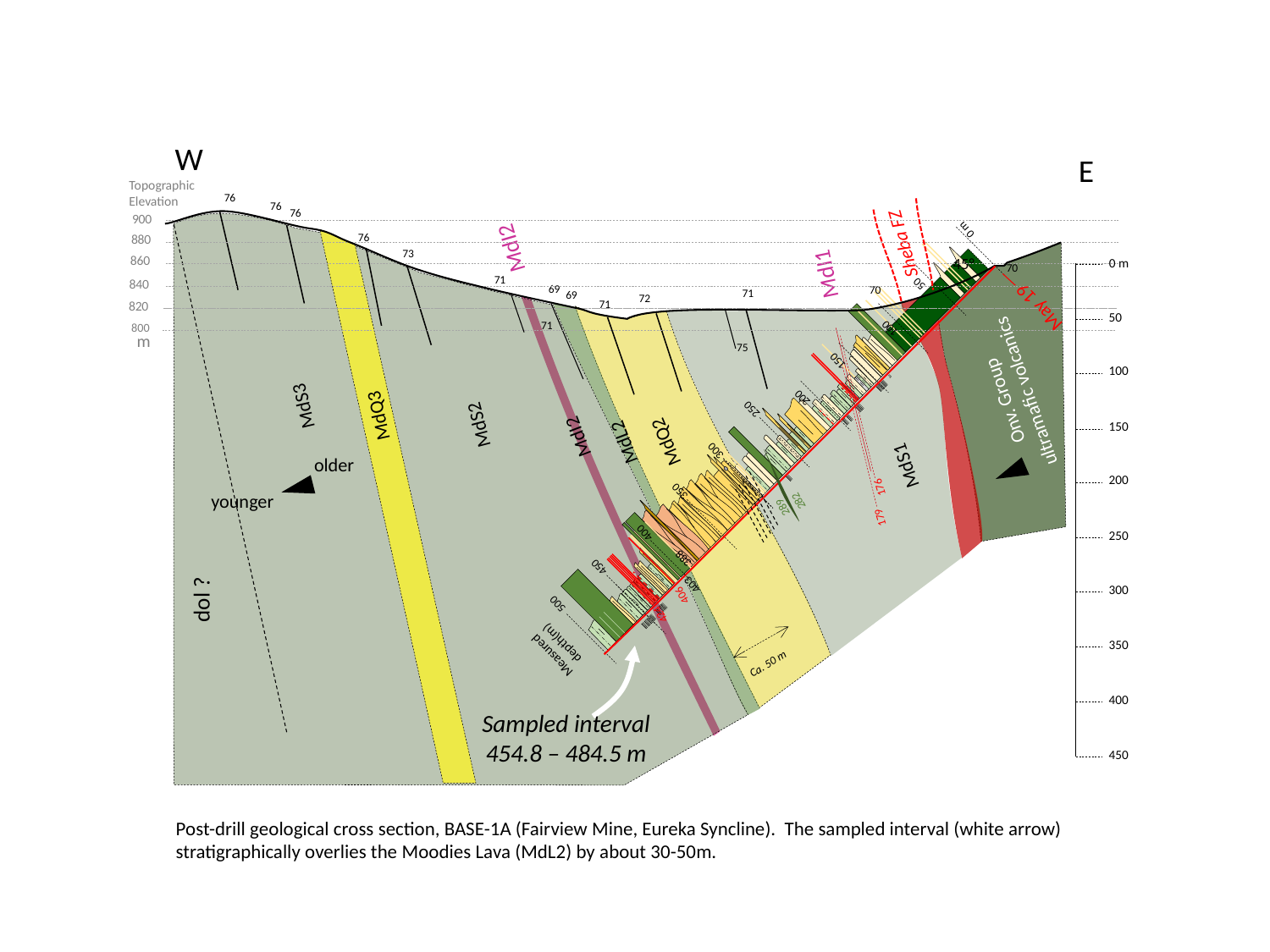

Appendix D – Geological Cross Sections
W
E
Topographic
Elevation
900
880
860
840
820
800
m
76
76
76
0 m
50
100
150
200
250
300
176
350
282
289
179
400
388
450
403
406
500
424
Measured depth(m)
76
Sheba FZ
MdI2
73
45°
0 m
50
100
150
200
250
300
350
400
450
MdI1
70
71
May 19
69
70
71
69
72
71
71
75
Onv. Group
 ultramafic volcanics
MdS3
MdQ3
MdS2
MdI2
MdL2
MdQ2
older
MdS1
younger
dol ?
Ca. 50 m
Sampled interval 454.8 – 484.5 m
Post-drill geological cross section, BASE-1A (Fairview Mine, Eureka Syncline). The sampled interval (white arrow) stratigraphically overlies the Moodies Lava (MdL2) by about 30-50m.
