# Supplementary material for: Magmatic and thermally produced reactive phosphorus 3.2 billion years ago and its implications for early life
Source: Commun Earth Environ. 2025 Nov 13;6(1):895. doi: 10.1038/s43247-025-02824-x (PMC12615253; doi:10.1038/s43247-025-02824-x)
Supplement: Supplementary file 5 — Supplementary Data SD2 [file 43247_2025_2824_MOESM5_ESM.zip › 1150 C_ Carbon Black.zip!/Mag-OM Exp CB control/2412191018-0-6-asb27/10/pdata/1/email_2412191018-0-6-asb27_10_1.pdf]

ƆM int. Exp CB control_1:10 EDNa 8 hr_10%D2O_15000 || 31P Observe with 1H decou

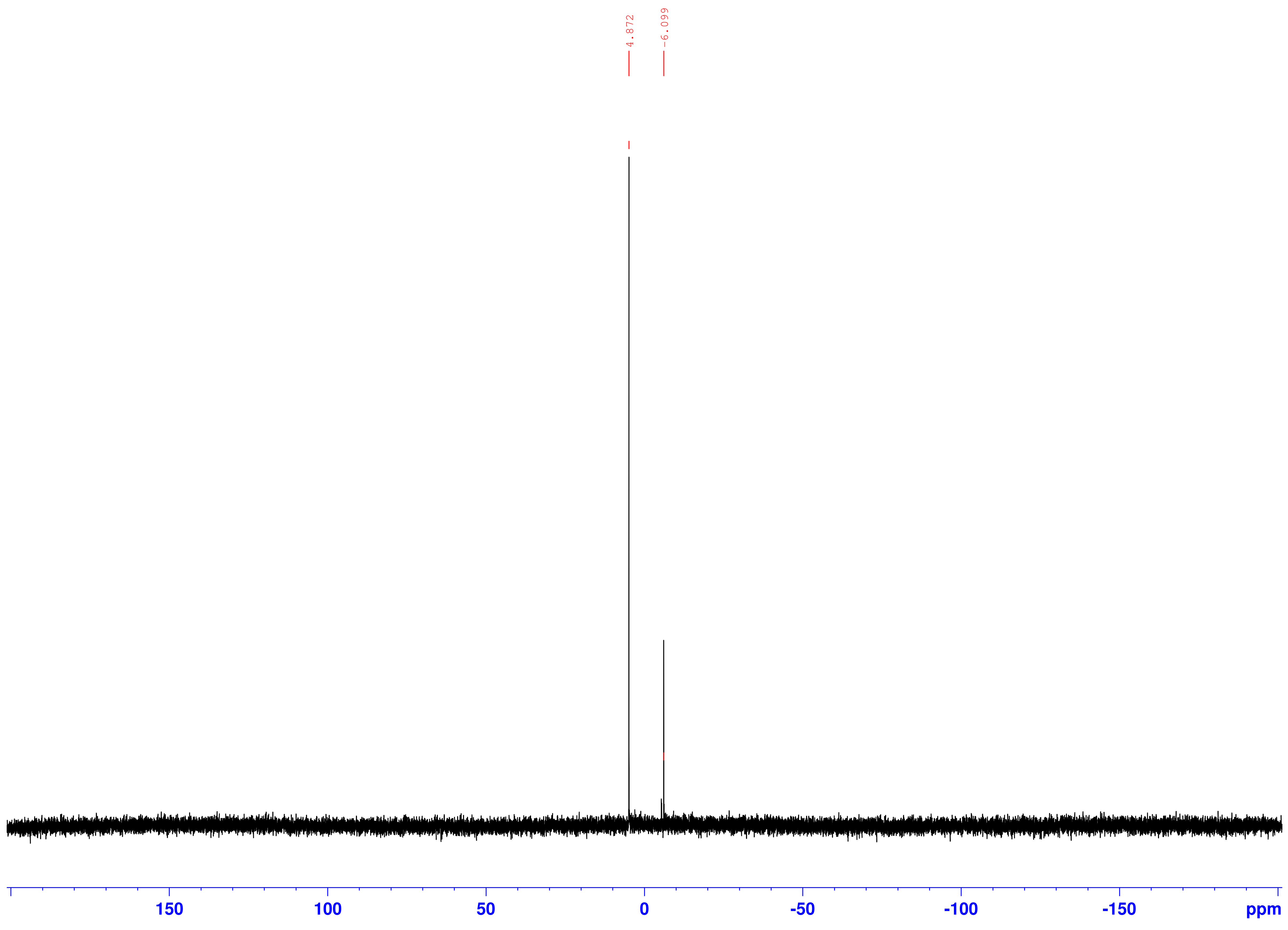


```
Current Data Parameters
NAME     2412191018-0-6-asb27
EXPNO                  10
PROCNO                  1

F2 - Acquisition Parameters
Date_            20241220
Time                3.15 h
INSTRUM             spect
PROBHD   Z130033_0010 (
PULPROG            zgpg30
TD                  65536
SOLVENT           H2O+D2O
NS                  15000
DS                      4
SWH             81521.742 Hz
FIDRES           2.487846 Hz
AQ              0.4019541 sec
RG                    128
DW                  6.133 usec
DE                  18.00 usec
TE                  298.0 K
D1             1.50000000 sec
D11            0.03000000 sec
TD0                     1
SFO1          202.4563350 MHz
NUC1                  31P
P0                   4.00 usec
P1                  12.00 usec
PLW1          48.00000000 W
SFO2          500.1320005 MHz
NUC2                   1H
CPDPRG[2          waltz65
PCPD2               80.00 usec
PLW2          17.01099968 W
PLW12          0.41532001 W
PLW13          0.20890000 W

F2 - Processing parameters
SI                  65536
SF            202.4563350 MHz
WDW                    EM
SSB                     0
LB                   2.00 Hz
GB                      0
PC                   1.40
```
