# Supplementary material for: Magmatic and thermally produced reactive phosphorus 3.2 billion years ago and its implications for early life
Source: Commun Earth Environ. 2025 Nov 13;6(1):895. doi: 10.1038/s43247-025-02824-x (PMC12615253; doi:10.1038/s43247-025-02824-x)
Supplement: Supplementary file 5 — Supplementary Data SD2 [file 43247_2025_2824_MOESM5_ESM.zip › 1150 C_ Carbon Black.zip!/Mag-OM Exp CB2 (Mg-PO4)/2406211755-0-11-asb27/10/pdata/1/email_2406211755-0-11-asb27_10_1.pdf]

action_Exp CB2(Mg-PO4)_1:10 EDNa_8hr_10% D2O_15000 scan || 31P Observe with 1

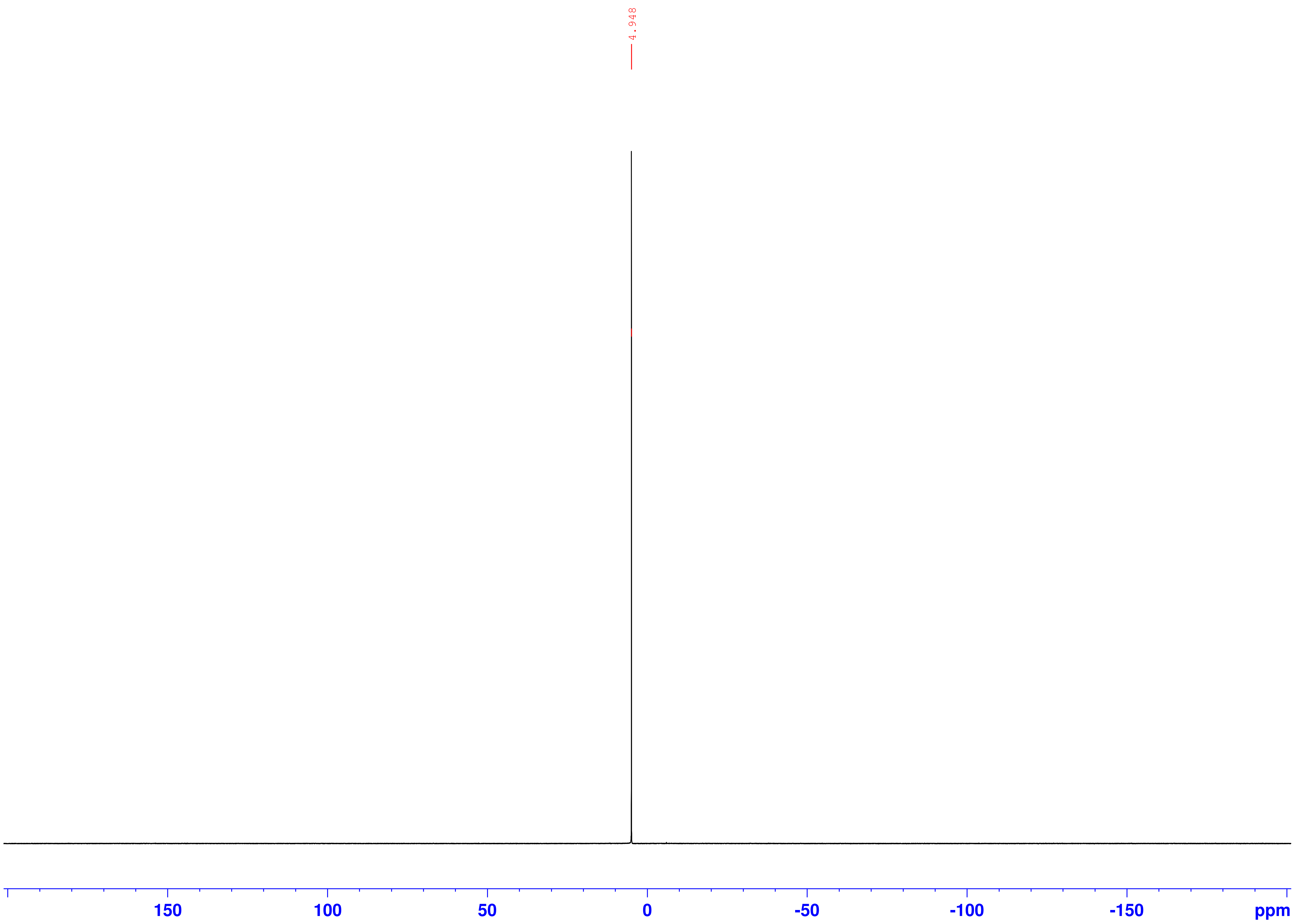


```
Current Data Parameters
NAME      2406211755-0-11-asb27
EXPNO                10
PROCNO                1

F2 - Acquisition Parameters
Date_          20240622
Time               4.12 h
INSTRUM           spect
PROBHD   Z130033_0010 (
PULPROG          zgpg30
TD                65536
SOLVENT         H2O+D2O
NS                15000
DS                    4
SWH           81521.742 Hz
FIDRES         2.487846 Hz
AQ            0.4019541 sec
RG                  128
DW                6.133 usec
DE                18.00 usec
TE                298.2 K
D1           1.50000000 sec
D11          0.03000000 sec
TD0                   1
SFO1        202.4563350 MHz
NUC1                31P
P0                 4.00 usec
P1                12.00 usec
PLW1        48.00000000 W
SFO2        500.1320005 MHz
NUC2                 1H
CPDPRG[2        waltz65
PCPD2             80.00 usec
PLW2        14.00000000 W
PLW12        0.31500000 W
PLW13        0.15843999 W

F2 - Processing parameters
SI                65536
SF          202.4563350 MHz
WDW                  EM
SSB                   0
LB                 2.00 Hz
GB                    0
PC                 1.40
```
