# Supplementary material for: Magmatic and thermally produced reactive phosphorus 3.2 billion years ago and its implications for early life
Source: Commun Earth Environ. 2025 Nov 13;6(1):895. doi: 10.1038/s43247-025-02824-x (PMC12615253; doi:10.1038/s43247-025-02824-x)
Supplement: Supplementary file 5 — Supplementary Data SD2 [file 43247_2025_2824_MOESM5_ESM.zip › 1150 C_OM and Controls.zip!/Mag-OM Exp 5_SiO2+OM+Mg-phos/10/pdata/1/email_2406101931-0-10-asb27_10_1.pdf]

**M interaction_Exp 5_1:10 EDNa_8 hr_10% D2O_15000 scan || 31P Observe with 1H de**

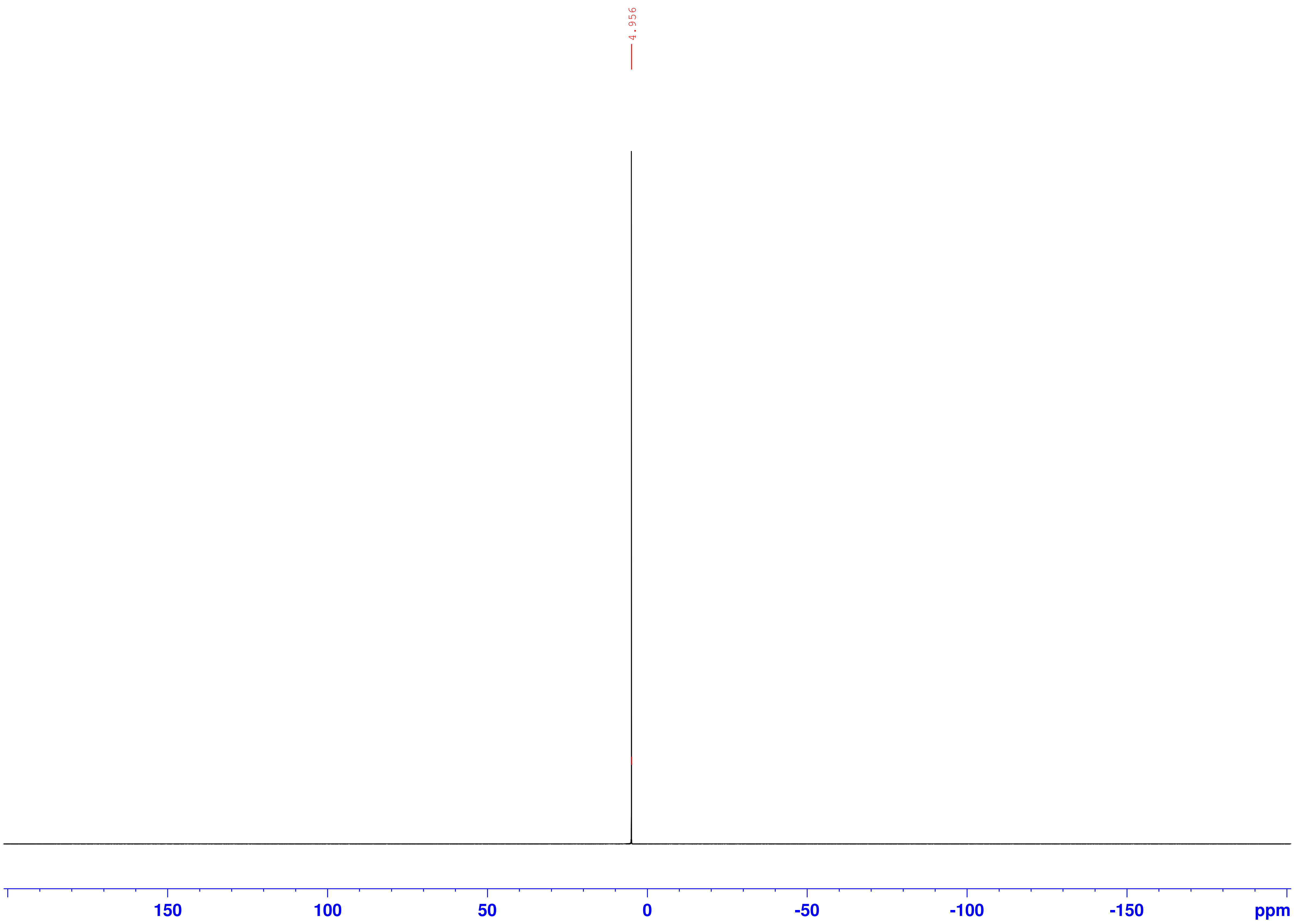


```
Current Data Parameters
NAME       2406101931-0-10-asb27
EXPNO                 10
PROCNO                 1

F2 - Acquisition Parameters
Date_           20240611
Time                4.49 h
INSTRUM            spect
PROBHD    Z130033_0010 (
PULPROG          zgpg30
TD                 65536
SOLVENT          H2O+D2O
NS                 15000
DS                     4
SWH            81521.742 Hz
FIDRES          2.487846 Hz
AQ             0.4019541 sec
RG                   128
DW                 6.133 usec
DE                 18.00 usec
TE                 298.2 K
D1            1.50000000 sec
D11           0.03000000 sec
TD0                    1
SFO1         202.4563350 MHz
NUC1                 31P
P0                  4.00 usec
P1                 12.00 usec
PLW1         48.00000000 W
SFO2         500.1320005 MHz
NUC2                  1H
CPDPRG[2          waltz65
PCPD2              80.00 usec
PLW2         14.00000000 W
PLW12         0.31500000 W
PLW13         0.15843999 W

F2 - Processing parameters
SI                 65536
SF           202.4563350 MHz
WDW                   EM
SSB                    0
LB                  2.00 Hz
GB                     0
PC                  1.40
```
