# Supplementary material for: Magmatic and thermally produced reactive phosphorus 3.2 billion years ago and its implications for early life
Source: Commun Earth Environ. 2025 Nov 13;6(1):895. doi: 10.1038/s43247-025-02824-x (PMC12615253; doi:10.1038/s43247-025-02824-x)
Supplement: Supplementary file 5 — Supplementary Data SD2 [file 43247_2025_2824_MOESM5_ESM.zip › 1150 C_OM and Controls.zip!/Mag-OM Exp 6_SiO2+Viv/2403150942-0-35-asb27/10/pdata/1/email_2403150942-0-35-asb27_10_1.pdf]

ı-OM interaction Exp 6_1:10 16 hour_10%D2O_4000 scan || 31P Observe with 1H decc

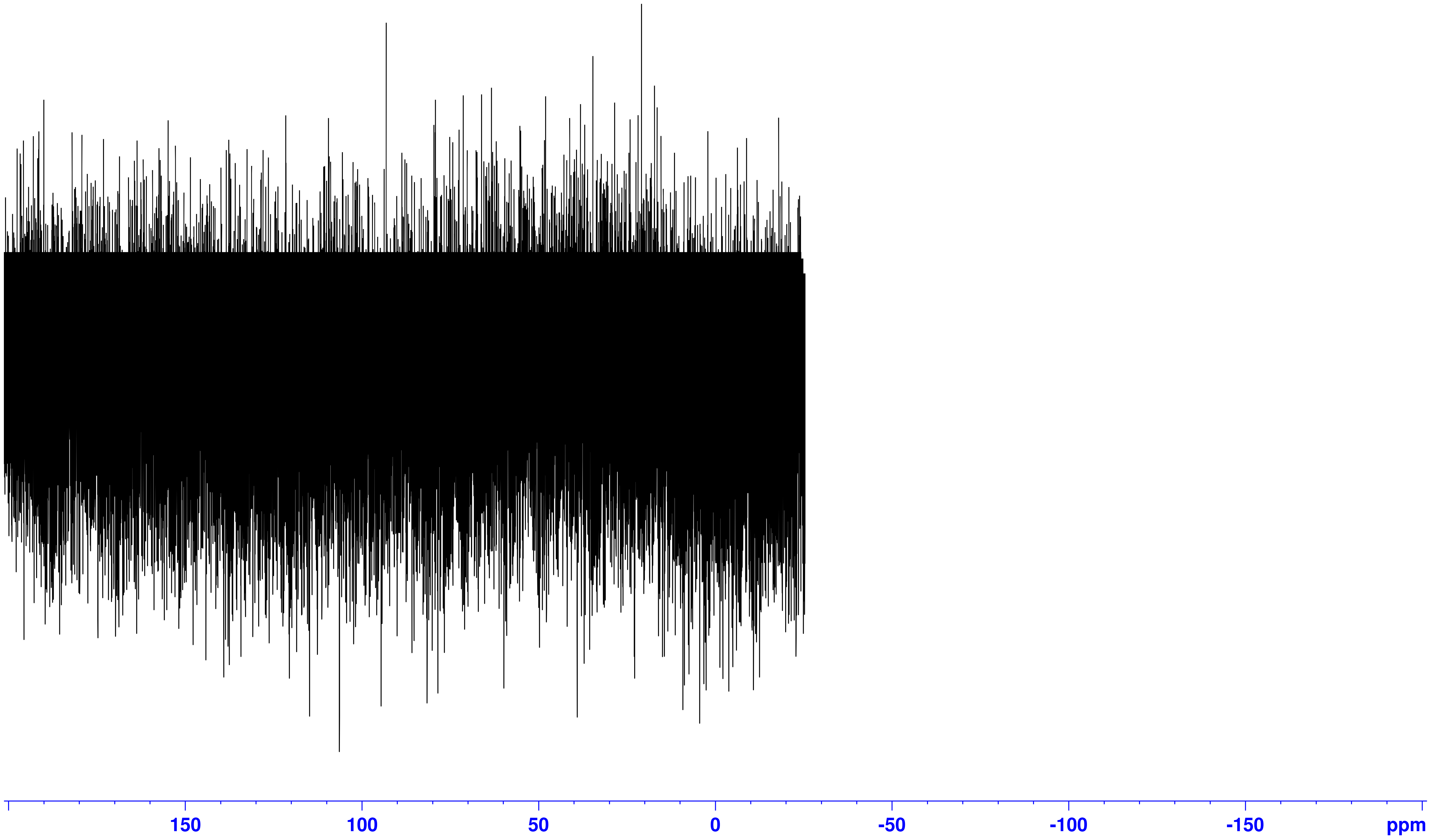

Current Data Parameters

NAME	2403150942-0-35-asb27	
EXPNO	10	
PROCNO	1	

F2 - Acquisition Parameters

Date_	20240315	
Time	12.00	h
INSTRUM	spect	
PROBHD	Z130033_0010 (	
PULPROG	zgpg30	
TD	65536	
SOLVENT	H2O+D2O	
NS	4000	
DS	4	
SWH	81521.742	Hz
FIDRES	2.487846	Hz
AQ	0.4019541	sec
RG	128	
DW	6.133	usec
DE	18.00	usec
TE	298.2	K
D1	1.50000000	sec
D11	0.03000000	sec
TD0	1	
SFO1	202.4563350	MHz
NUC1	31P	
P0	4.00	usec
P1	12.00	usec
PLW1	48.00000000	W
SFO2	500.1320005	MHz
NUC2	1H	
CPDPRG[2	waltz65	
PCPD2	80.00	usec
PLW2	14.00000000	W
PLW12	0.31500000	W
PLW13	0.15843999	W

F2 - Processing parameters

SI	65536	
SF	202.4563350	MHz
WDW	EM	
SSB	0	
LB	2.00	Hz
GB	0	
PC	1.40	
