# Supplementary material for: Magmatic and thermally produced reactive phosphorus 3.2 billion years ago and its implications for early life
Source: Commun Earth Environ. 2025 Nov 13;6(1):895. doi: 10.1038/s43247-025-02824-x (PMC12615253; doi:10.1038/s43247-025-02824-x)
Supplement: Supplementary file 5 — Supplementary Data SD2 [file 43247_2025_2824_MOESM5_ESM.zip › 1150 C_OM and Controls.zip!/Mag-OM Exp 7_SiO2+Amph Fe-PO4/2403150942-0-36-asb27/10/pdata/1/email_2403150942-0-36-asb27_10_1.pdf]

ı-OM interaction Exp 7_1:10 16 hour_10%D2O_4000 scan || 31P Observe with 1H decc

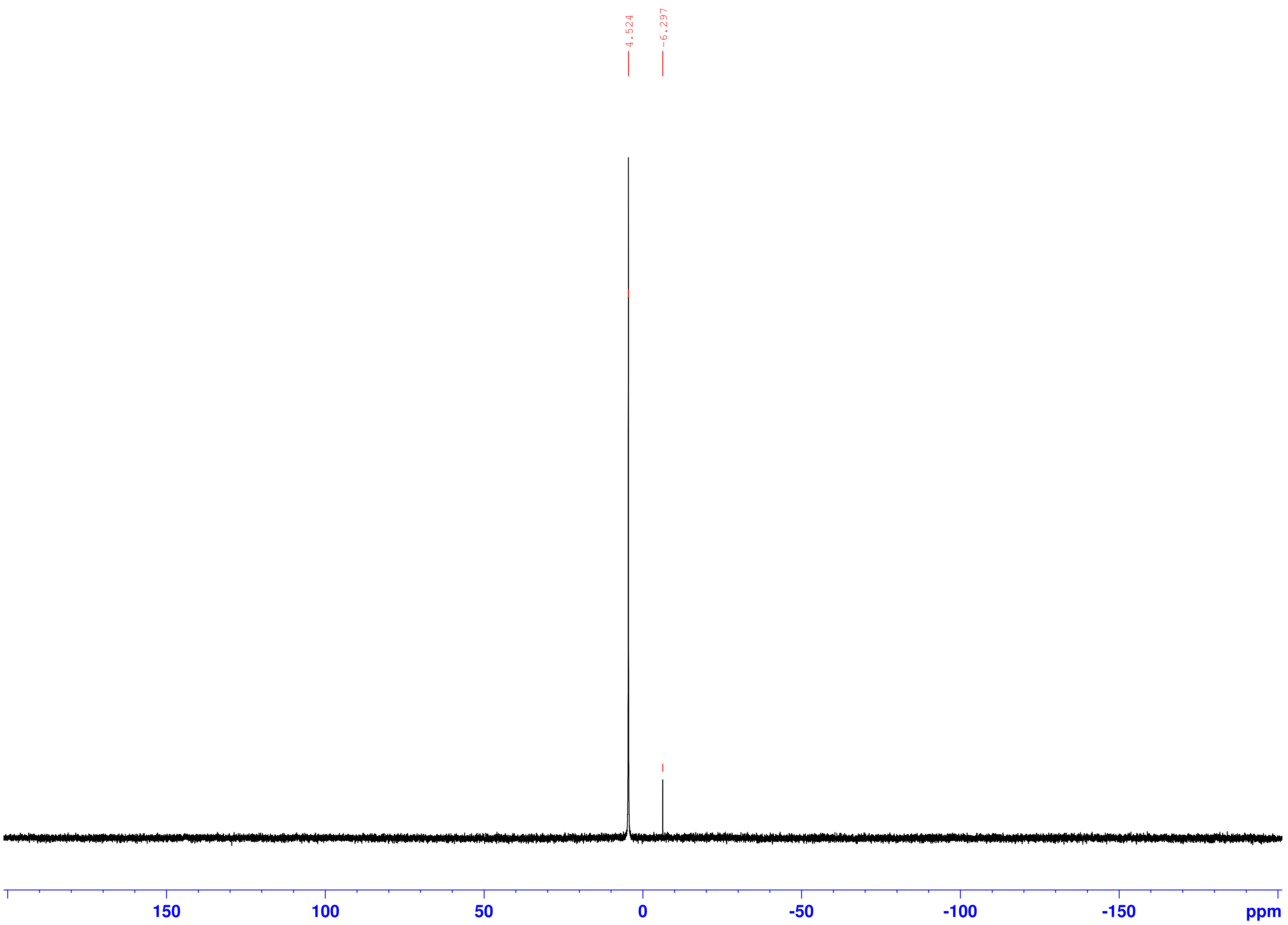


```
Current Data Parameters
NAME      2403150942-0-36-asb27
EXPNO                    10
PROCNO                    1

F2 - Acquisition Parameters
Date_              20240315
Time                  14.14 h
INSTRUM               spect
PROBHD   Z130033_0010 (
PULPROG              zgpg30
TD                    65536
SOLVENT             H2O+D2O
NS                     4000
DS                        4
SWH               81521.742 Hz
FIDRES             2.487846 Hz
AQ                0.4019541 sec
RG                      128
DW                    6.133 usec
DE                    18.00 usec
TE                    298.1 K
D1               1.50000000 sec
D11              0.03000000 sec
TD0                       1
SFO1            202.4563350 MHz
NUC1                    31P
P0                     4.00 usec
P1                    12.00 usec
PLW1            48.00000000 W
SFO2            500.1320005 MHz
NUC2                     1H
CPDPRG[2            waltz65
PCPD2                 80.00 usec
PLW2            14.00000000 W
PLW12            0.31500000 W
PLW13            0.15843999 W

F2 - Processing parameters
SI                    65536
SF              202.4563350 MHz
WDW                      EM
SSB                       0
LB                     2.00 Hz
GB                        0
PC                     1.40
```
