# Supplementary material for: Magmatic and thermally produced reactive phosphorus 3.2 billion years ago and its implications for early life
Source: Commun Earth Environ. 2025 Nov 13;6(1):895. doi: 10.1038/s43247-025-02824-x (PMC12615253; doi:10.1038/s43247-025-02824-x)
Supplement: Supplementary file 5 — Supplementary Data SD2 [file 43247_2025_2824_MOESM5_ESM.zip › 1150 C_OM and Controls.zip!/Mag-OM Exp 8_SiO2+Apa/10/pdata/1/email_2406071848-0-13-asb27_10_1.pdf]

Mag-OM_Exp 8_1:10_8hr_10%D2O_15000 scan || 31P Observe with 1H decoupling

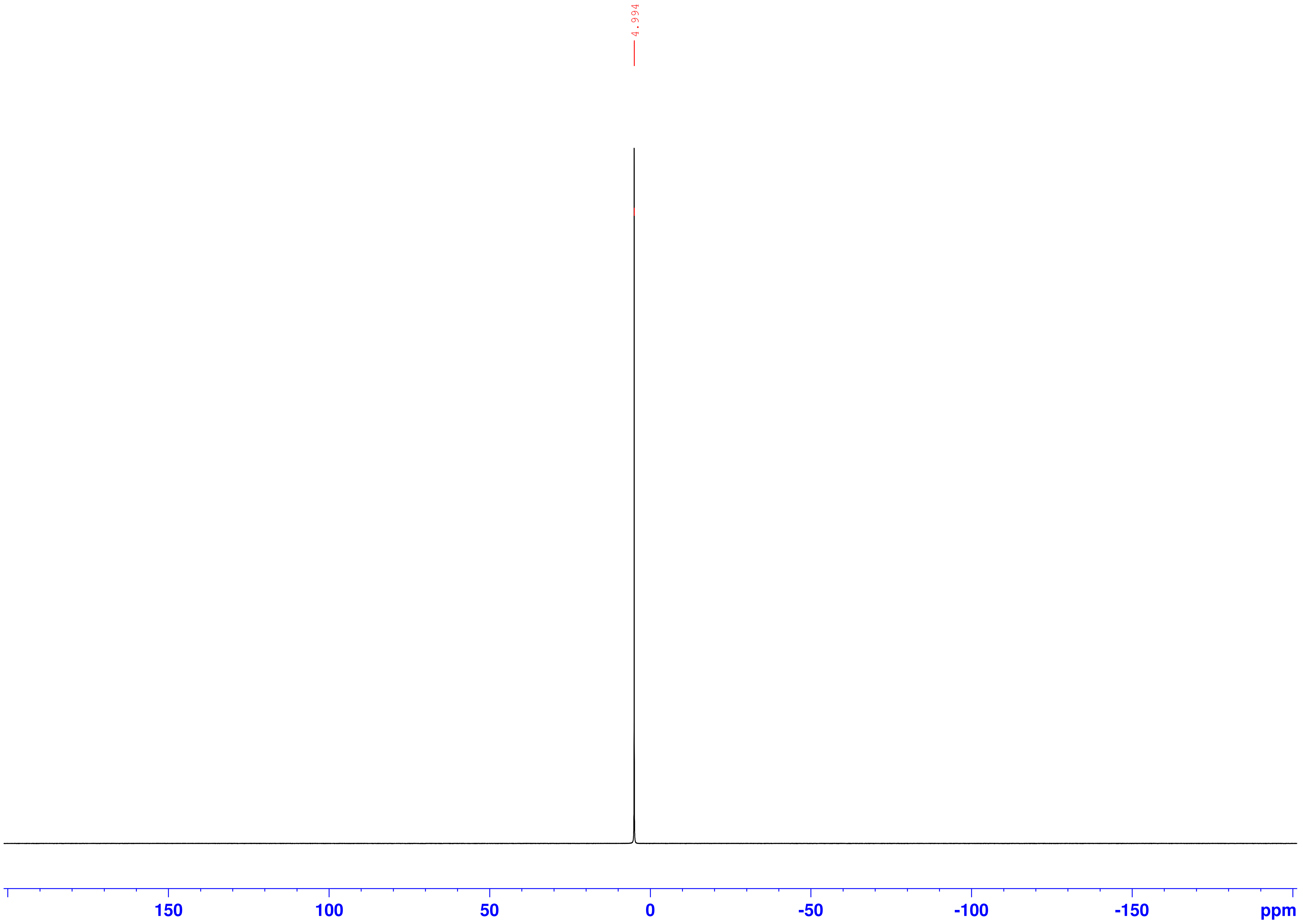


```
Current Data Parameters
NAME     2406071848-0-13-asb27
EXPNO                   10
PROCNO                   1

F2 - Acquisition Parameters
Date_             20240608
Time                  4.12 h
INSTRUM              spect
PROBHD   Z130033_0010 (
PULPROG             zgpg30
TD                   65536
SOLVENT            H2O+D2O
NS                   15000
DS                       4
SWH             81521.742 Hz
FIDRES           2.487846 Hz
AQ              0.4019541 sec
RG                     128
DW                   6.133 usec
DE                   18.00 usec
TE                   298.2 K
D1             1.50000000 sec
D11            0.03000000 sec
TD0                      1
SFO1          202.4563350 MHz
NUC1                   31P
P0                    4.00 usec
P1                   12.00 usec
PLW1          48.00000000 W
SFO2          500.1320005 MHz
NUC2                    1H
CPDPRG[2           waltz65
PCPD2                80.00 usec
PLW2          14.00000000 W
PLW12          0.31500000 W
PLW13          0.15843999 W

F2 - Processing parameters
SI                   65536
SF            202.4563350 MHz
WDW                     EM
SSB                      0
LB                    2.00 Hz
GB                       0
PC                    1.40
```
