# Supplementary material for: Magmatic and thermally produced reactive phosphorus 3.2 billion years ago and its implications for early life
Source: Commun Earth Environ. 2025 Nov 13;6(1):895. doi: 10.1038/s43247-025-02824-x (PMC12615253; doi:10.1038/s43247-025-02824-x)
Supplement: Supplementary file 5 — Supplementary Data SD2 [file 43247_2025_2824_MOESM5_ESM.zip › 1300 C_ Carbon Black.zip!/Mag-OM Exp CBHT2 (apa)/2406221016-0-13-asb27/10/pdata/1/email_2406221016-0-13-asb27_10_1.pdf]

nt_Exp CBHT2(apa)_1:10 EDNa_12 hr_10% D2O_15000 scan || 31P Observe with 1H d

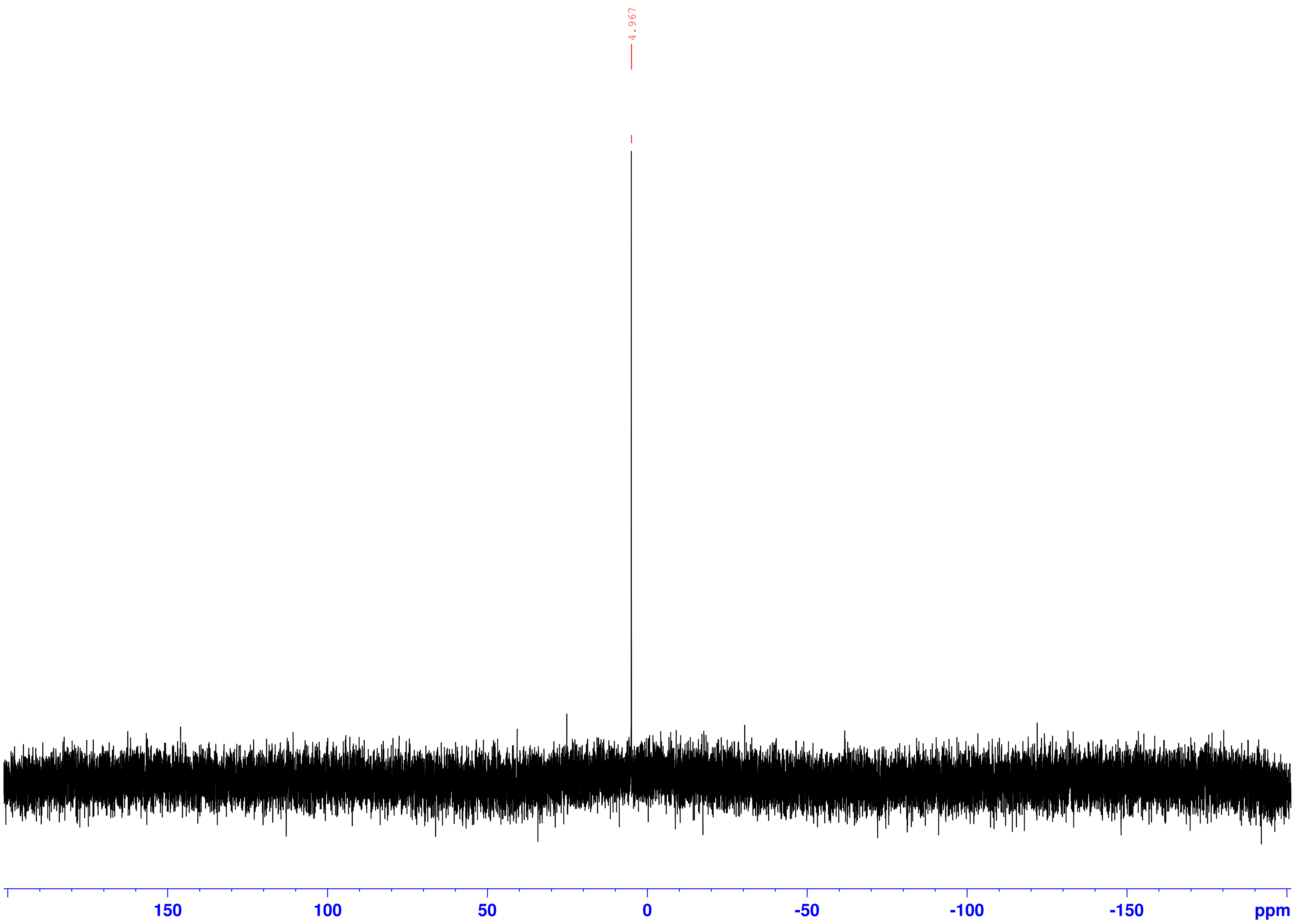


```
Current Data Parameters
NAME     2406221016-0-13-asb27
EXPNO                       10
PROCNO                       1

F2 - Acquisition Parameters
Date_              20240622
Time                  19.40 h
INSTRUM               spect
PROBHD   Z130033_0010 (
PULPROG              zgpg30
TD                    65536
SOLVENT             H2O+D2O
NS                    15000
DS                        4
SWH              81521.742 Hz
FIDRES            2.487846 Hz
AQ               0.4019541 sec
RG                      128
DW                    6.133 usec
DE                    18.00 usec
TE                    298.2 K
D1               1.50000000 sec
D11              0.03000000 sec
TD0                       1
SFO1         202.4563350 MHz
NUC1                    31P
P0                     4.00 usec
P1                    12.00 usec
PLW1         48.00000000 W
SFO2         500.1320005 MHz
NUC2                     1H
CPDPRG[2            waltz65
PCPD2                 80.00 usec
PLW2         14.00000000 W
PLW12         0.31500000 W
PLW13         0.15843999 W

F2 - Processing parameters
SI                    65536
SF           202.4563350 MHz
WDW                      EM
SSB                       0
LB                     2.00 Hz
GB                        0
PC                     1.40
```
