# Supplementary material for: Magmatic and thermally produced reactive phosphorus 3.2 billion years ago and its implications for early life
Source: Commun Earth Environ. 2025 Nov 13;6(1):895. doi: 10.1038/s43247-025-02824-x (PMC12615253; doi:10.1038/s43247-025-02824-x)
Supplement: Supplementary file 5 — Supplementary Data SD2 [file 43247_2025_2824_MOESM5_ESM.zip › 1300 C_ Carbon Black.zip!/Mag-OM Exp CBHTApaFe/2407061558-0-16-asb27/10/pdata/1/email_2407061558-0-16-asb27_10_1.pdf]

Exp CBHTApaFe_1:10 EDNa_12 hr_10% D2O_15000 scan || 31P Observe with 1H dec

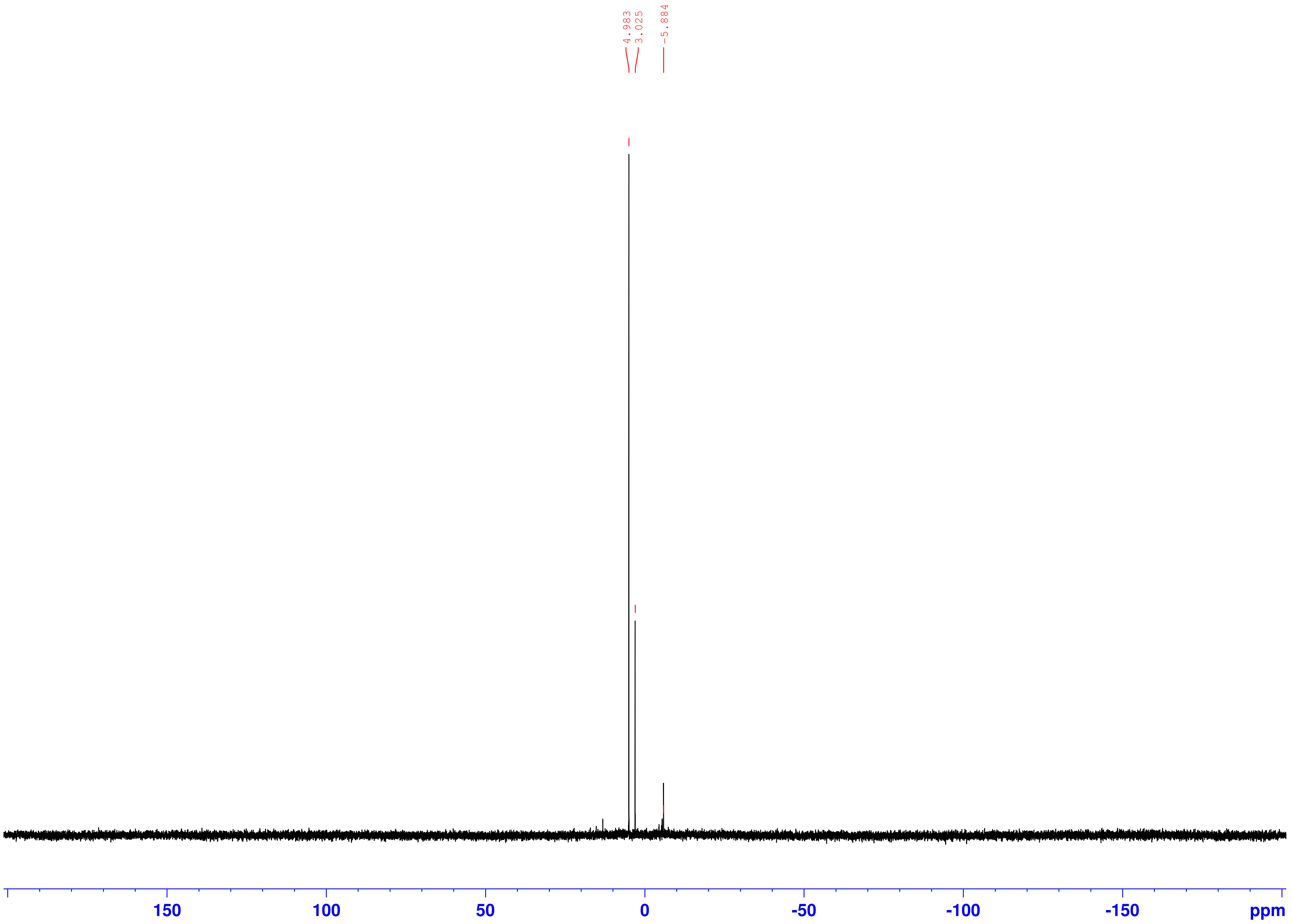

Current Data Parameters
NAME 2407061558-0-16-asb27
EXPNO 10
PROCNO 1

F2 - Acquisition Parameters
Date_ 20240707
Time 3.15 h
INSTRUM spect
PROBHD Z130033_0010 (
PULPROG zgpg30
TD 65536
SOLVENT H2O+D2O
NS 15000
DS 4
SWH 81521.742 Hz
FIDRES 2.487846 Hz
AQ 0.4019541 sec
RG 128
DW 6.133 usec
DE 18.00 usec
TE 298.2 K
D1 1.50000000 sec
D11 0.03000000 sec
TD0 1
SFO1 202.4563350 MHz
NUC1 31P
P0 4.00 usec
P1 12.00 usec
PLW1 48.00000000 W
SFO2 500.1320005 MHz
NUC2 1H
CPDPRG[2 waltz65
PCPD2 80.00 usec
PLW2 14.00000000 W
PLW12 0.31500000 W
PLW13 0.15843999 W

F2 - Processing parameters
SI 65536
SF 202.4563350 MHz
WDW EM
SSB 0
LB 2.00 Hz
GB 0
PC 1.40
