# Supplementary material for: Magmatic and thermally produced reactive phosphorus 3.2 billion years ago and its implications for early life
Source: Commun Earth Environ. 2025 Nov 13;6(1):895. doi: 10.1038/s43247-025-02824-x (PMC12615253; doi:10.1038/s43247-025-02824-x)
Supplement: Supplementary file 5 — Supplementary Data SD2 [file 43247_2025_2824_MOESM5_ESM.zip › 1300 C_ Carbon Black.zip!/Mag-OM Exp CBHTApaFe3O4/2407060849-0-8-asb27/10/pdata/1/email_2407060849-0-8-asb27_10_1.pdf]

CBHTApaFe3O4_1:10 EDNa 12 hr_5% NaOH_10%D2O_15000 || 31P Observe with 1H

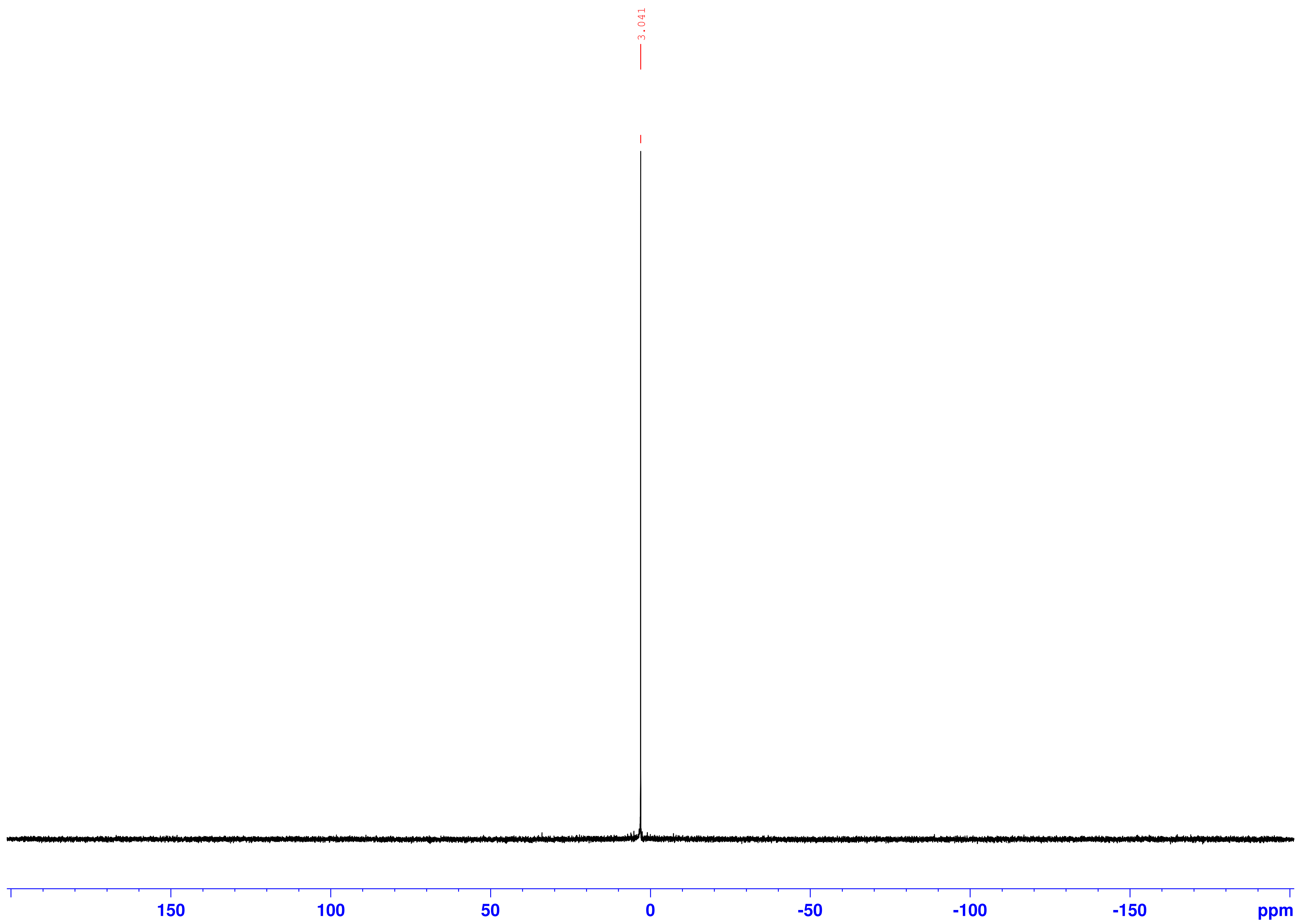

Current Data Parameters

NAME	2407060849-0-8-asb27
EXPNO	10
PROCNO	1

F2 - Acquisition Parameters

Date_	20240706
Time	18.04 h
INSTRUM	spect
PROBHD	Z130033_0010 (
PULPROG	zgpg30
TD	65536
SOLVENT	H2O+D2O
NS	15000
DS	4
SWH	81521.742 Hz
FIDRES	2.487846 Hz
AQ	0.4019541 sec
RG	128
DW	6.133 usec
DE	18.00 usec
TE	298.1 K
D1	1.50000000 sec
D11	0.03000000 sec
TD0	1
SFO1	202.4563350 MHz
NUC1	31P
P0	4.00 usec
P1	12.00 usec
PLW1	48.00000000 W
SFO2	500.1320005 MHz
NUC2	1H
CPDPRG[2	waltz65
PCPD2	80.00 usec
PLW2	14.00000000 W
PLW12	0.31500000 W
PLW13	0.15843999 W

F2 - Processing parameters

SI	65536
SF	202.4563350 MHz
WDW	EM
SSB	0
LB	2.00 Hz
GB	0
PC	1.40
